# Social vulnerability and health determinants of Intrinsic Capacity in Mexican older adults: Evidence from the Mexican Health and Aging Study round 2021

**DOI:** 10.1371/journal.pone.0353903

**Published:** 2026-08-07

**Authors:** Miriam Teresa López-Teros, Fabiola Yocupicio Medrano, Karla Animas Mijangos, Omar Yaxmehen Bello-Chavolla, Alberto Jose Mimenza-Alvarado, Sara Gloria Aguilar-Navarro

**Affiliations:** 1 Department of Applied Nutrition and Nutrition Education, Instituto Nacional de Ciencias Médicas y Nutrición Salvador Zubirán, Mexico City, Mexico; 2 Department of Geriatrics, Instituto Nacional de Ciencias Médicas y Nutrición Salvador Zubirán, Mexico City, Mexico; 3 Department of Clinical Epidemiology, Instituto Nacional de Geriatría, Mexico City, Mexico; Hospital Infantil de México Federico Gomez: Hospital Infantil de Mexico Federico Gomez, MEXICO

## Abstract

**Background:**

Population aging has increased interest in Intrinsic Capacity (IC), defined as the composite of physical and mental abilities that support healthy aging. Because declines in IC precede disability, IC is a useful framework for characterizing individuals with greater vulnerability in later life.

**Methods:**

We conducted a cross-sectional analysis of the 2021 Mexican Health and Aging Study, a nationally representative survey of adults aged 50 years and older. IC was assessed across five domains using domain-specific impairment, number of impaired domains, and a continuous latent IC score derived from factor analysis. Ordinal logistic regression models were used to examine associations with sociodemographic characteristics, health conditions, and a Social Vulnerability Index (SVI) constructed using a cumulative deficit approach.

**Findings:**

The sample included 13,403 adults (mean age 65.9 years [SD 9.8]; 57.3% women). IC impairment was highly prevalent: 67.1% had impairment in two or more domains, whereas only 12.5% had no impaired domains. Lower IC was associated with increasing age (≥80 years: OR 3.31; 95% CI 1.10–10.02) and poorer health, including multimorbidity (OR 2.29; 95% CI 1.44–3.64) and poor self-rated health (OR 1.69; 95% CI 1.24–2.28). Lower IC was further associated with social disadvantage, including lower educational attainment (OR 2.33; 95% CI 1.69–3.22) and food insecurity (OR 1.51; 95% CI 1.06–2.16), with higher social vulnerability associated with lower IC (β −0.46; 95% CI −0.51 to −0.41).

**Interpretation:**

IC impairment is highly prevalent among Mexican adults aged 50 years and older and is strongly associated with social vulnerability and modifiable health factors, supporting IC as an integrative framework to identify vulnerable individuals and inform intervention strategies.

## Introduction

Aging is a lifelong process marked by progressive structural and functional changes that determine health across the lifespan [1]. As populations age, interest in healthy aging has shifted from disease-based definitions toward functional approaches [2]. In 2015, the World Health Organization (WHO) defined healthy aging as the process of developing and maintaining functional ability, which arises from Intrinsic Capacity (IC), environmental characteristics, and their interaction [3].

IC encompasses all the mental and physical capacities that an individual can draw upon [3]. It is a multidimensional construct influenced by demographic, socioeconomic, and health-related factors [4,5]. The clinical and practical relevance of IC lies in its strong association with health and functional status in older adults [6,7]. Higher IC has been associated with greater functional independence and survival, whereas lower IC has been linked to frailty, falls, hospitalization, institutionalization, polypharmacy, and increased mortality [2,5–10]. IC is typically assessed across five domains—locomotion, cognition, psychological, vitality, and sensory—often guided by the WHO’s Integrated Care for Older People (ICOPE) framework [2,11–13].

Several studies report that 55%–77% of community-dwelling older adults present impairment in IC [14–18]. However, prevalence estimates vary across populations and settings. Evidence from a previous study conducted in Mexico, based on a secondary analysis of the Mexican Health and Aging Study (MHAS), highlighted a substantial burden of IC impairment among adults aged ≥50 years, with approximately 88% of participants presenting impairment in at least one IC domain [19]. Prevalence increased with age and exceeded 90% among the oldest age groups [18].

IC is influenced by multiple factors, including age-related biological changes, health-related behaviors, disease burden, and broader socioeconomic and physical conditions experienced throughout life [5,19–21]. Although the ICOPE framework facilitates IC assessment in primary care, its domain-focused approach may not fully capture these broader determinants of IC [4,22,23]. In this context, Social Determinants of Health (SDH) and social vulnerability provide a useful framework for understanding how broader social and contextual factors may be associated with differences in IC.

SDH are the broader conditions in which individuals are born, grow, live, work, and age, encompassing structural factors such as economic systems, social policies, and the distribution of power and resources that influence health opportunities and outcomes [24–26]. Social vulnerability refers to a state of reduced resilience and increased susceptibility to adverse consequences during crises or stressful events, arising from accumulated social disadvantages [24,27]. Together, SDH and social vulnerability represent closely interconnected concepts: SDH comprise the broader structural and contextual conditions that influence health and well-being, whereas social vulnerability reflects the resulting level of risk and reduced adaptive capacity associated with unfavorable conditions [24,27]. Social disadvantages often coexist and cluster within individuals and populations [25,28,29]. Exposure to multiple social conditions throughout life may contribute to inequalities in IC, understood as the uneven distribution of physical and mental capacities across population groups resulting from differences in social, economic, and environmental circumstances rather than chronological aging alone [30].

Given the multifactorial nature of IC, updated evidence is needed to better understand the factors associated with its impairment, particularly in low- and middle-income countries. Using nationally representative data from Mexico, this study assesses the associations of individual-level health and social factors, and cumulative social vulnerability, with IC.

## Materials and Methods

### Study design and data source

The Mexican Health and Aging Study (MHAS) is a nationally representative longitudinal survey of adults aged 50 years and older designed to collect detailed sociodemographic, health, and functional information to examine ageing and its determinants. Established in 2001, the study has conducted multiple follow-up waves and includes a broad range of domains, including social, economic, demographic, and health-related variables. MHAS is supported by the US National Institutes of Health/National Institute on Aging (grant R01AG018016) and by the National Institute of Statistics and Geography (INEGI) in Mexico. For this study, data from the 2021 wave were used. The data were accessed for research purposes between 01/03/2025 and 31/12/2025 and are publicly available upon registration through the MHAS website (www.mhasweb.org).

The present study is a secondary analysis of publicly available, anonymized data from the Mexican Health and Aging Study (MHAS). As no identifiable participant information was accessed and no new data were collected, the study was exempt from ethics committee review in accordance with institutional and national regulations.

### Sample selection

**Fig 1:**
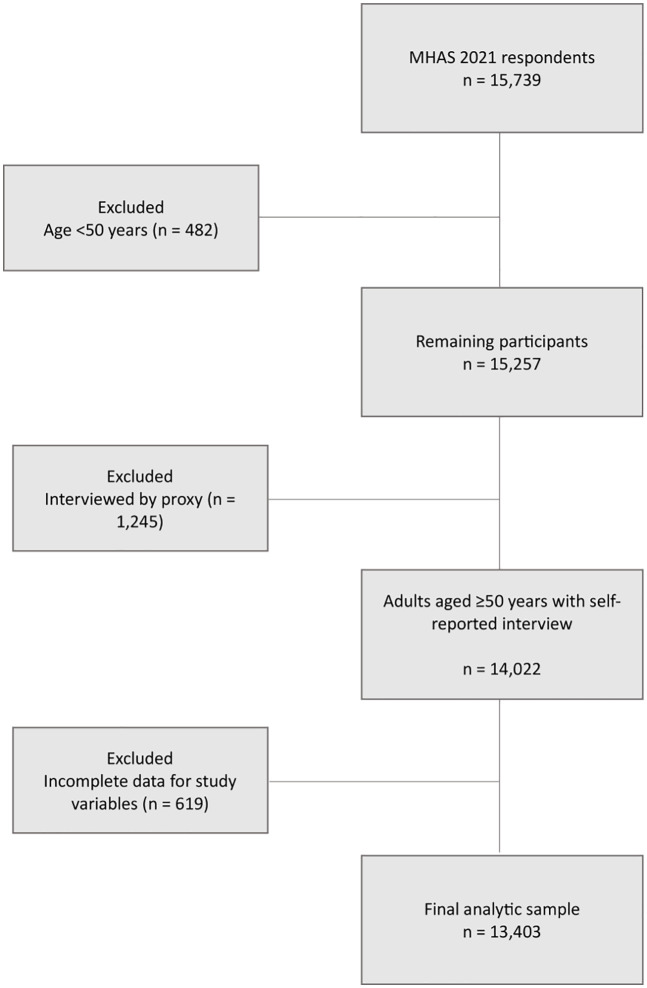
Study Sample Selection Flowchart. Of the 15,739 respondents, those aged <50 years (n = 482), interviewed by proxy (n = 1,245), or with incomplete data (n = 619) were excluded, resulting in a final analytic sample of 13,403 adults aged ≥50 years.

The 2021 MHAS wave included 15,739 respondents. Participants younger than 50 years (n = 482) and those interviewed by proxy (n = 1,245) were excluded, yielding 14,022 adults aged ≥50 years. Participants with incomplete data (n = 619) were further excluded, resulting in a final analytic sample of 13,403 individuals (Fig 1).

### Measures

#### Intrinsic capacity.

A composite index of IC was constructed according to the WHO ICOPE framework, drawing on methodologies previously applied in population-based longitudinal cohort studies [31–33]. IC was operationalized across five domains (cognitive, psychological, sensory, mobility, and vitality) using proxy measures available in the MHAS. The corresponding MHAS questions, variables, and operational definitions are presented in S1 Table.

**1) Psychological:** depressive symptoms were assessed using a modified nine-item version of the Center for Epidemiologic Studies Depression (CES-D) scale included in the MHAS. This version comprises dichotomous (yes/no) items referring to experiences in the past week, including feeling depressed, having difficulty performing activities, restless sleep, feeling happy, loneliness, enjoyment of life, sadness, fatigue, and low energy. In accordance with a clinical validation study, a binary variable was constructed, classifying participants as having depressive symptoms if the summed score was ≥ 5 [34].
**2) Sensorial impairment:** This variable was derived from self-reported vision (Respondent’s vision with glasses) and hearing (Respondent uses hearing/auditory device). Vision impairment was coded as 1 if the participant reported impaired vision and 0 otherwise. Hearing impairment was coded as 1 if the participant reported impaired hearing and 0 otherwise.
**3) Vitality:** In line with prior work, and consistent with the two screening questions proposed in the WHO ICOPE guidelines, this domain was defined using weight change compared to two years ago and reduced food intake over the past two years [13,18]. Participants were classified as impaired (1) if either or both conditions were present, and as not impaired (0) otherwise.
**4) Cognitive impairment:** was assessed using orientation (day, month, and year) and verbal recall. The latter variable was assessed as impaired if participants did not recall at least three words in the immediate eight-word recall test. Orientation and memory items were combined into a binary indicator; impairment (1) was assigned if deficits were present in either domain.
**5) Locomotor impairment:** this domain was defined based on three self-reported functional limitations due to a health problem, including difficulty walking several blocks, difficulty climbing flights of stairs, and difficulty rising from a chair. Participants were classified as impaired (1) if they reported a limitation in at least one of these activities, and as not impaired (0) otherwise.

A composite index of IC was constructed following the WHO ICOPE framework. An Exploratory Factor Analysis (EFA) using scree plot inspection and oblique (promax) rotation was first performed to identify the underlying structure. Subsequently, a Confirmatory Factor Analysis (CFA) within a Structural Equation Modeling (SEM) framework was applied to assess model fit and domain interrelations. The final model included five correlated domains: cognitive, psychological, sensory, mobility, and vitality—each represented by multiple observed indicators. Detailed standardized factor loadings for all indicators are presented in S1 Fig. The model showed adequate goodness of fit, and standardized factor scores were generated and normalized (mean = 0, SD = 1) to produce a continuous IC variable. Higher IC scores reflected better preserved capacity, whereas lower scores indicated greater IC deficits. In addition, domain-specific binary indicators were derived to identify the presence or absence of impairment within each domain.

### Sociodemographic characteristics

Variables included sex, age, education, and marital status. Age was analyzed both continuously and in categories (50–59, 60–69, 70–79, ≥ 80 years). Education was classified as ≤7 or >7 years of schooling, and marital status as living with a partner (married or cohabiting), widowed, or single (never married, separated, or divorced). Additional socioeconomic factors included employment status (current labor force participation), pension receipt during the previous year, food insecurity (self-reported insufficient money to purchase food in the past two years), and healthcare affiliation (any public or private coverage vs none).

### Health determinants

Health determinants and clinical conditions were assessed using self-reported. Poor self-rated health was defined as reporting fair or poor health. Chronic conditions (hypertension, diabetes, osteoarthritis, acute myocardial infarction, pulmonary disease, neoplasms, and stroke) were coded as present or absent, and multimorbidity was defined as the presence of two or more chronic conditions. Falls at least one event in the past two years and sleep disturbances were defined as difficulty initiating or maintaining sleep.

### Lifestyle and behavioral factors

Alcohol consumption was coded as present (1) for current drinking, while smoking was coded as present (1) for current or former smokers. Leisure and social engagement were defined as participation in activities such as crafts, volunteering, educational activities, sports or social clubs, reading, puzzles or number games, or board games. Absence of leisure activities (1) was defined as no participation in any of these activities.

### Social vulnerability Index (SVI)

To capture cumulative social vulnerability beyond individual socioeconomic indicators, we constructed a Social Vulnerability Index (SVI) using a cumulative deficit approach, consistent with established frameworks on social determinants of health in older adults [26,30]. The index integrates indicators reflecting socioeconomic disadvantage, limited social protection, and material deprivation. Specifically, the SVI included low educational attainment, not currently working, lack of pension income, food insecurity, lack of health-care affiliation, and living alone. Each component was coded as a binary variable (0 = absence, 1 = presence), and the overall SVI score was calculated as the sum of deficits, ranging from 0 to 6, with higher scores indicating greater social vulnerability. For analytical purposes, the SVI was categorized into tertiles (low, intermediate, and high vulnerability) based on its distribution in the study population.

### Statistical analysis

Descriptive statistics were used to characterize the study population according to sociodemographic, health-related, and lifestyle variables. Continuous variables are presented as means and standard deviations (SD), and categorical variables as frequencies and percentages.

To comprehensively examine IC, we applied complementary analytical approaches that capture both clinically interpretable categories and the underlying continuous construct of IC. Participants were classified according to the number of impaired IC domains into three ordered groups: no impaired domains (Group 0), one impaired domain (Group 1), and two or more impaired domains (Group 2). Ordinal logistic regression was used to identify factors associated with greater IC impairment, and results are presented as odds ratios (ORs) with 95% confidence intervals (CIs). Covariates included demographic characteristics (age group and sex); socioeconomic characteristics (marital status, education, employment, pension receipt, food insecurity, and health-care affiliation); health and clinical factors (self-rated health, falls, multimorbidity, and sleep disturbances); and lifestyle behaviors (smoking and alcohol consumption).

To evaluate associations with the continuous latent IC score derived from factor analysis, multivariable linear regression models were fitted using the same covariates to ensure comparability across analyses. Results are reported as β coefficients and 95% confidence intervals (CIs).

For comparative purposes, the continuous IC score was additionally categorized into tertiles (high, intermediate, and low), and multinomial logistic regression models were estimated using the high IC tertile as the reference category. Results are presented as relative risk ratios (RRRs) and 95% CIs.

Finally, associations between the SVI and IC were examined using multivariable linear regression models with the continuous IC score as the outcome and SVI tertiles as the main exposure. Models were adjusted for age group, sex, self-rated health, falls, multimorbidity, sleep disturbances, smoking, and alcohol consumption, with results reported as β coefficients and 95% CIs.

To assess the robustness of the SVI, a sensitivity analysis was conducted excluding employment status from the index and repeating all SVI analyses. In addition, multicollinearity among covariates included in the multivariable models was evaluated using variance inflation factors (VIFs). Results of the sensitivity and multicollinearity analyses are presented in S2 Table and S3 Table, respectively.

All analyses accounted for the complex survey design of MHAS using sampling weights, clustering, and stratification variables to obtain nationally representative estimates and appropriate standard errors.

All statistical tests were two-sided, and statistical significance was defined as a p-value < 0.05. Analyses were conducted using Stata version 14.0 (Stata Corp LLC, College Station, TX, USA) and R (R Foundation for Statistical Computing, Vienna, Austria).

## Results

The analytical sample included 13,403 community-dwelling adults aged ≥50 years (mean age 65.9 ± 9.8 years), of whom 57.3% were women. Most participants were living with a partner (66.1%), were not currently employed (60.7%), did not receive a pension (80.9%), and had ≤ 7 years of education (55.9%). Food insecurity was reported by 26.8% of participants, and 23.1% lacked healthcare affiliation. Regarding health characteristics, more than half of participants reported fair or poor self-rated health (55.6%), 40.1% had experienced at least one fall in the previous two years, and 40.4% reported sleep disturbances. Hypertension (47.1%) and diabetes (26.6%) were the most prevalent chronic conditions. Based on IC, 12.5% of participants had no impaired domains (Group 0), 20.4% had impairment in one domain (Group 1), and 67.1% presented impairment in two or more domains (Group 2). The prevalence of IC impairments varied across domains, with sensory impairment being the most frequent (56.7%), followed by cognitive (47.1%), locomotor (43.1%), psychological (40%), and vitality impairment (29.8%). Mean standardized IC scores differed significantly across groups, ranging from 1.19 (SD = 0.21) in Group 0 to 0.75 (SD = 0.41) in Group 1 and −0.45 (SD = 0.89) in Group 2 (Table 1).

**Table 1:** Participant Characteristics by Number of Impaired IC Domains.

Variable	Total Sample (n = 13,403)	Group 0 (n = 1,676)	Group 1 (n = 2,732)	Group 2 (n = 8,995)	p-value
**Sociodemographic Characteristics**					
Age, years (mean ± SD)	65.9 ± 9.8	61.6 ± 7.5	63.7 ± 8.4	67.5 ± 10.1	<0.001
Age group, years (n, %)					
50–59	4,492 (33.5)	815 (48.6)	1,098 (40.2)	2,579 (28.7)	
60–69	4,125 (30.8)	595 (35.5)	930 (34.0)	2,600 (28.9)	
70–79	3,432 (25.6)	232 (13.8)	587 (21.5)	2,613 (29.1)	
≥80	1,354 (10.1)	34 (2.0)	117 (4.3)	1,203 (13.4)	<0.001
Female sex (n, %)	7,674 (57.3)	834 (49.8)	1,452 (53.6)	5,388 (59.9)	<0.001
Marital status (n, %)					
Single¹	1,769 (13.3)	251 (15.1)	392 (14.5)	1,126 (12.6)	
Widowed	2,747 (20.7)	155 (9.4)	426 (15.7)	2,166 (24.2)	
With partner (married or cohabiting)	8,784 (66.1)	1,252 (75.5)	1,890 (69.8)	5,642 (63.2)	<0.001
≤7 years of education (n, %)	7,489 (55.9)	481 (28.7)	1,162 (42.5)	5,846 (64.9)	<0.001
Not currently employed (n, %)	8,126 (60.7)	775 (46.4)	1,399 (51.2)	5,952 (66.3)	<0.001
Not receiving a pension (n, %)	10,825 (80.9)	1,309 (78.2)	2,146 (78.6)	7,370 (82.0)	<0.001
Food insecurity (n, %)	3,577 (26.8)	292 (17.5)	583 (21.4)	2,702 (30.2)	<0.001
No health care affiliation² (n, %)	3,090 (23.2)	566 (33.8)	800 (29.3)	1,724 (19.2)	<0.001
**Health, clinical, and lifestyle characteristics**					
Self-rated health (fair/poor) (n, %)	7,376 (55.6)	731 (43.9)	1,304 (48.2)	5,341 (60.0)	<0.001
History of falls (last 2 years) (n, %)	5,374 (40.1)	394 (23.5)	870 (31.9)	4,110 (45.7)	<0.001
Hypertension (n, %)	6,312 (47.1)	534 (31.9)	1,074 (39.3)	4,704 (52.3)	<0.001
Diabetes (n, %)	3,558 (26.6)	248 (14.8)	542 (19.9)	2,768 (30.8)	<0.001
Osteoarthritis (n, %)	1,521 (11.4)	71 (4.2)	158 (5.8)	1,292 (14.4)	<0.001
Acute myocardial infarction (n, %)	472 (3.5)	15 (0.9)	66 (2.4)	391 (4.4)	<0.001
Pulmonary disease (n, %)	664 (4.9)	29 (1.7)	81 (2.9)	554 (6.2)	<0.001
Stroke (n, %)	255 (1.9)	12 (0.7)	21 (0.8)	222 (2.5)	<0.001
Sleep disturbances (n, %)	5,407 (40.4)	242 (14.4)	667 (24.4)	4,498 (50)	<0.001
Current alcohol consumption (n, %)	3,776 (28.2)	603 (35.9)	908 (33.2)	2,265 (25.2)	<0.001
Smoking (current or former) (n, %)	4,939 (36.9)	623 (37.2)	1,027 (37.6)	3,289 (36.6)	0.602
No leisure activities (n, %)	4,140 (30.9)	346 (20.6)	656 (24.0)	3,138 (34.9)	<0.001
**Domain-specific impairment and intrinsic capacity scores**					
Cognitive impairment (%)	6,309 (47.1)	----	790 (28.9)	5,519 (61.4)	<0.001
Psychological impairment (%)	5,366 (40.0)	----	324 (11.9)	5,042 (56.1)	<0.001
Sensory impairment (%)	7,601 (56.7)	----	798 (29.2)	6,803 (75.6)	<0.001
Locomotor impairment (%)	5,775 (43.1)	----	406 (14.9)	5,369 (59.7)	<0.001
Vitality impairment (%)	3,989 (29.8)	----	414 (15.2)	3,575 (39.7)	<0.001
Intrinsic capacity score	0.00 (1.0)	1.19 (0.2)	0.75 (0.4)	−0.45 (0.9)	<0.001

*^1^Single includes participants who reported being single, divorced, or separated. ² Health care affiliation includes IMSS, ISSSTE, INSABI, PEMEX, and private health services. Continuous variables are presented as mean (SD) and categorical variables as n (%). P-values were obtained using one-way ANOVA for continuous variables and χ² tests for categorical variables.*

**Fig 2:**
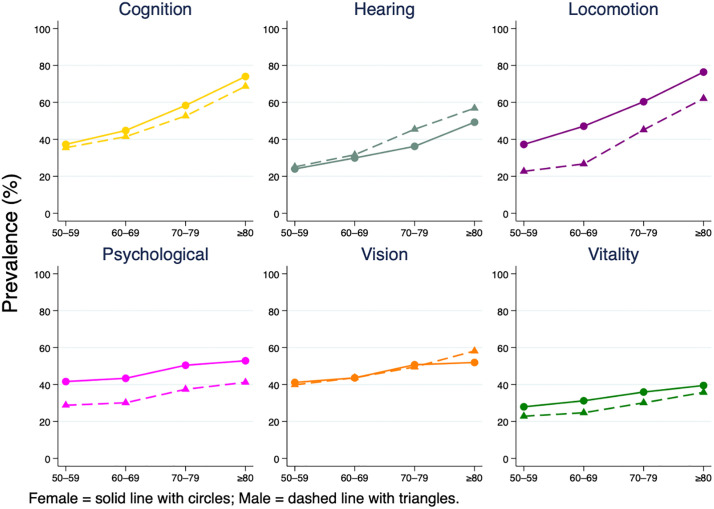
Prevalence of IC domain impairments by sex and age group. Lines represent the prevalence (%) of impairments in cognition, sensory, locomotion, psychological and vitality among adults aged 50 years and older. Solid lines with circles indicate women, and dashed lines with triangles indicate men.

Marked differences were observed in the distribution of IC domains by age and sex (Fig. 2). The prevalence of impairments generally increased with advancing age across all domains. Cognitive and locomotor impairments showed the most pronounced age-related increases, particularly among women. Sensory also became progressively more frequent in older age groups. Psychological was consistently more prevalent among women than men across most age groups, while vitality impairment showed a more modest increase with age.

In the ordinal logistic regression model, increasing age was associated with higher odds of presenting greater IC deficits. Compared with adults aged 50–59 years, those aged 70–79 years (OR = 1.8; 95% CI: 1.6–2.1, p < 0.001) and ≥80 years (OR = 4.0; 95% CI: 3.3–4.9, p < 0.001) had significantly higher odds of worse IC, whereas the association for those aged 60–69 years was not statistically significant (OR = 1.1; 95% CI: 1.0–1.2, p = 0.085). Lower educational attainment (≤7 years) was associated with higher odds of IC deficits (OR = 2.1; 95% CI: 2.0–2.3, p < 0.001). Food insecurity was also associated with higher odds of worse IC (OR = 1.5; 95% CI: 1.4–1.7, p < 0.001), as were not being currently employed (OR = 1.2; 95% CI: 1.1–1.3, p < 0.001), not receiving a pension (OR = 1.3; 95% CI: 1.1–1.4, p < 0.001), and lacking healthcare affiliation (OR = 1.4; 95% CI: 1.3–1.5, p < 0.001). Poor self-rated health (OR = 1.4; 95% CI: 1.3–1.5, p < 0.001), history of falls (OR = 1.6; 95% CI: 1.4–1.7, p < 0.001), multimorbidity (OR = 1.9; 95% CI: 1.7–2.1, p < 0.001), and sleep disturbances (OR = 3.2; 95% CI: 2.9–3.5, p < 0.001) were associated with higher odds of worse IC. Smoking (current or former) was also associated with greater odds of IC deficits (OR = 1.2; 95% CI: 1.1–1.3, p < 0.001), whereas current alcohol consumption was associated with lower odds of worse IC (OR = 0.9; 95% CI: 0.8–1.0, p = 0.002). No significant associations were observed for sex or being single/divorced/separated, although widowed participants had slightly higher odds of worse IC (OR = 1.2; 95% CI: 1.1–1.4, p = 0.002) (Table 2).

**Table 2:** Factors associated with intrinsic capacity deficits (ordinal logistic regression).

Variable	OR (95% CI)	p-value
Age group, years		
60–69	1.09 (0.99–1.20)	0.085
70–79	1.82 (1.61–2.05)	**<0.001**
≥80	4.02 (3.27–4.94)	**<0.001**
Female sex	0.99 (0.90–1.09)	0.864
Marital status		
Widowed	1.20 (1.07–1.35)	**0.002**
Single/Divorced/Separated	1.05 (0.94–1.18)	0.369
≤7 years of education	2.12 (1.95–2.31)	**<0.001**
Not currently employed	1.19 (1.09–1.31)	**<0.001**
Not receiving a pension	1.26 (1.14–1.40)	**<0.001**
Food insecurity	1.50 (1.37–1.65)	**<0.001**
No health care affiliation	1.40 (1.27–1.53)	**<0.001**
Poor self-rated health	1.42 (1.31–1.53)	**<0.001**
History of falls	1.57 (1.44–1.71)	**<0.001**
Multimorbidity (≥2 chronic conditions)	1.89 (1.71–2.10)	**<0.001**
Sleep disturbances	3.22 (2.94–3.52)	**<0.001**
Current alcohol consumption	0.87 (0.79–0.95)	**0.002**
Smoking (current or former)	1.20 (1.10–1.31)	**<0.001**

*Abbreviations: OR, odds ratio; CI, confidence interval.*

When IC was modeled as a standardized continuous score, multivariable linear regression analyses yielded consistent results. Compared with participants aged 50–59 years, those aged 60–69 years (β = −0.07; 95% CI: −0.10 to −0.04), 70–79 years (β = −0.34; 95% CI: −0.38 to −0.30), and ≥80 years (β = −0.76; 95% CI: −0.82 to −0.70) had lower IC scores (all p < 0.001). Female sex (β = −0.08; 95% CI: −0.11 to −0.05), widowed status (β = −0.12; 95% CI: −0.16 to −0.08), lower educational attainment (β = −0.29; 95% CI: −0.32 to −0.26), not being currently employed (β = −0.15; 95% CI: −0.18 to −0.11), not receiving a pension (β = −0.09; 95% CI: −0.13 to −0.06), food insecurity (β = −0.18; 95% CI: −0.21 to −0.14), and lack of healthcare affiliation (β = −0.18; 95% CI: −0.21 to −0.15) were associated with lower IC scores. Poor self-rated health (β = −0.24; 95% CI: −0.27 to −0.21), history of falls (β = −0.25; 95% CI: −0.27 to −0.22), multimorbidity (β = −0.36; 95% CI: −0.40 to −0.33), and sleep disturbances (β = −0.51; 95% CI: −0.54 to −0.48) were also associated with lower IC scores (all p < 0.001). Current alcohol consumption was positively associated with IC (β = 0.08; 95% CI: 0.05 to 0.12), whereas smoking (current or former) was associated with lower IC scores (β = −0.06; 95% CI: −0.10 to −0.03). No significant association was observed for being single/divorced/separated (Table 3).

**Table 3:** Factors associated with intrinsic capacity score: multivariable analysis.

Variable	β	95% CI	p-value
Age group, years			
60–69	−0.071	−0.104, −0.037	**<0.001**
70–79	−0.338	−0.380, −0.296	**<0.001**
≥80	−0.761	−0.823, −0.699	**<0.001**
Female sex	−0.081	−0.114, −0.047	**<0.001**
Marital status			
Widowed	−0.115	−0.155, −0.075	**<0.001**
Single/Divorced/Separated	−0.020	−0.060, 0.020	0.327
≤7 years of education	−0.285	−0.315, −0.255	**<0.001**
Not currently employed	−0.147	−0.180, −0.114	**<0.001**
Not receiving a pension	−0.093	−0.130, −0.056	**<0.001**
Food insecurity	−0.175	−0.207, −0.143	**<0.001**
No health care affiliation	−0.179	−0.211, −0.148	**<0.001**
Poor self-rated health	−0.240	−0.268, −0.212	**<0.001**
History of falls	−0.245	−0.274, −0.215	**<0.001**
Multimorbidity	−0.362	−0.396, −0.327	**<0.001**
Sleep disturbances	−0.509	−0.539, −0.479	**<0.001**
Current alcohol consumption	0.083	0.052, 0.115	**<0.001**
Smoking (current or former)	−0.064	−0.095, −0.033	**<0.001**

When IC was categorized into tertiles, multinomial regression analyses showed consistent gradients across sociodemographic, socioeconomic, and health-related factors. Increasing age, female sex, lower educational attainment, lack of pension income, food insecurity, lack of healthcare affiliation, poor self-rated health, history of falls, multimorbidity, sleep disturbances, and smoking were associated with a higher relative risk of belonging to lower IC categories. Several of these associations were stronger for low versus high IC than for intermediate versus high IC, particularly for older age groups, multimorbidity, and sleep disturbances. Sleep disturbances showed the strongest association with low IC (RRR = 5.24; 95% CI: 4.69–5.85; p < 0.001). In contrast, current alcohol consumption was associated with a lower relative risk of belonging to both the low and intermediate IC groups (Table 4).

**Table 4:** Relative risks for low and intermediate intrinsic capacity, with high capacity as reference.

Variable	Low vs High RRR (95% CI)	p-value	Intermediate vs High RRR (95% CI)	p-value
Age group, years				
60–69	1.28 (1.12–1.46)	<0.001	1.14 (1.02–1.27)	**0.024**
70–79	3.03 (2.58–3.55)	<0.001	1.90 (1.65–2.18)	**<0.001**
≥80	10.32 (8.06–13.23)	<0.001	3.06 (2.41–3.88)	**<0.001**
Female sex	1.43 (1.26–1.63)	<0.001	1.33 (1.19–1.48)	**<0.001**
Marital status				
Widowed	1.36 (1.18–1.57)	<0.001	1.12 (0.98–1.28)	0.097
Single/Divorced/Separated	1.08 (0.92–1.27)	0.355	1.11 (0.97–1.26)	0.127
≤7 years of education	2.58 (2.30–2.89)	<0.001	1.84 (1.67–2.03)	**<0.001**
Not currently employed	1.56 (1.38–1.77)	<0.001	1.08 (0.98–1.20)	0.124
Not receiving a pension	1.28 (1.11–1.47)	<0.001	1.16 (1.03–1.31)	**0.015**
Food insecurity	1.75 (1.55–1.97)	<0.001	1.29 (1.16–1.43)	**<0.001**
No health care affiliation	2.05 (1.80–2.34)	<0.001	1.38 (1.24–1.53)	**<0.001**
Poor self-rated health	2.19 (1.97–2.44)	<0.001	1.27 (1.16–1.39)	**<0.001**
History of falls	2.14 (1.92–2.38)	<0.001	1.27 (1.16–1.40)	**<0.001**
Multimorbidity	3.14 (2.77–3.56)	<0.001	1.51 (1.34–1.70)	**<0.001**
Sleep disturbances	5.24 (4.69–5.85)	<0.001	2.40 (2.17–2.65)	**<0.001**
Current alcohol consumption	0.77 (0.68–0.87)	<0.001	0.89 (0.81–0.99)	**0.025**
Smoking (current or former)	1.27 (1.13–1.42)	<0.001	1.15 (1.04–1.27)	**0.005**

*Abbreviations: RRR, Relative Risk Ratio; CI, confidence interval.*

Incorporating the SVI as both a categorical and continuous variable revealed a graded association characterized by progressively lower IC scores across increasing levels of social vulnerability. When modeled as a continuous variable, higher SVI scores were significantly associated with lower IC (β = −0.06; 95% CI: −0.08 to −0.05, p < 0.001). Similarly, when SVI was categorized into tertiles, participants in the intermediate vulnerability tertile had lower IC scores (β = −0.24; 95% CI: −0.28 to −0.20, p < 0.001), whereas those in the highest vulnerability tertile exhibited an even greater reduction in IC (β = −0.45; 95% CI: −0.50 to −0.40, p < 0.001) compared with participants in the lowest vulnerability tertile (Table 5).

**Table 5:** Association between social vulnerability tertiles and intrinsic capacity score.

Variable	β coefficient	95% CI	p-value
Social Vulnerability Index (SVI)			
Low vulnerability (reference)	0.00	—	—
Intermediate vulnerability	−0.241	−0.279, −0.204	<0.001
High vulnerability	−0.448	−0.497, −0.400	<0.001
SVI score	−0.062	−0.076, −0.049	<0.001
Age group (years)			
50–59 (reference)	0.00	—	—
60–69	−0.112	−0.144, −0.079	<0.001
70–79	−0.415	−0.453, −0.377	<0.001
≥80	−0.856	−0.912, −0.799	<0.001
Female sex	−0.095	−0.127, −0.063	<0.001
Poor self-rated health	−0.154	−0.185, −0.124	<0.001
History of falls	−0.265	−0.295, −0.235	<0.001
Multimorbidity	−0.398	−0.432, −0.363	<0.001
Sleep disturbances	−0.538	−0.568, −0.508	<0.001
Current alcohol consumption	0.101	0.069, 0.133	<0.001
Smoking (current or former)	−0.060	−0.091, −0.029	<0.001

*Abbreviations: CI, confidence interval.*

**Fig 3:**
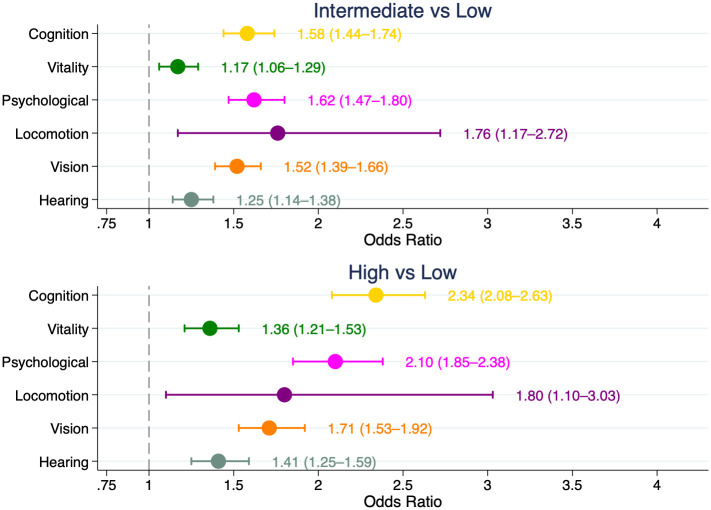
Association Between Social Vulnerability Tertiles and Intrinsic Capacity Domain Impairments. Compared with the low-vulnerability tertile (reference), individuals in the intermediate- and high-vulnerability tertiles had progressively higher odds of impairment across all domains, with the strongest associations observed for cognition, psychological capacity, and locomotion. Estimates were adjusted for sociodemographic and health-related covariates.

Domain-specific analyses showed differences across IC domains according to SVI tertiles. Compared with individuals in the low-vulnerability tertile (reference), those in the intermediate-vulnerability tertile had higher odds of impairment across all domains, particularly locomotion (OR 1.76; 95% CI 1.17–2.72), psychological (OR 1.62; 95% CI 1.47–1.80), and cognition (OR 1.58; 95% CI 1.44–1.74). Similar but stronger associations were observed among participants in the high-vulnerability tertile, with the largest effects for cognition (OR 2.34; 95% CI 2.08–2.63) and psychological (OR 2.10; 95% CI 1.85–2.38). Odds of sensory impairment, and vitality were also significantly higher among individuals with greater SVI. Estimates were adjusted for sociodemographic and health-related covariates (Fig 3).

Multicollinearity diagnostics showed low variance inflation factors across all covariates included in the multivariable models (VIF range: 1.04–1.78; mean VIF = 1.26), indicating no evidence of problematic multicollinearity (Supplementary S3 Table).

Supplementary Table S1 provides a detailed description of the operationalization of intrinsic capacity domains according to the WHO ICOPE framework using variables available in the Mexican Health and Aging Study. Supplementary S2 Fig presents the conceptual framework linking SD, social vulnerability, the SVI, and intrinsic capacity.

## Discussion

IC reflects the composite of physical and mental capacities that enable individuals to adapt to aging-related changes [6,12,13,35]. As declines in IC often precede overt disability, it provides a multidimensional framework for understanding functional aging beyond the presence of disease [1,6,10,36]. In this nationally representative sample, IC impairment was highly prevalent: over 67% of participants presented deficits in ≥2 domains, and only 12.5% retained full capacity. These estimates exceed those reported in high-income countries and are consistent with patterns observed in low- and middle-income settings [36–38]. These findings position IC not only as a clinical construct, but as a population-level indicator of structural inequality [28,36,38].

Advancing age was strongly associated with lower IC and greater multidomain impairment, consistent with the well-established inverse relationship between chronological age and functional reserve [6,10,36,39]. However, marked heterogeneity was observed, with a substantial proportion retaining full or near-full capacity, reinforcing the dynamic and modifiable nature of IC [6,36,40,41]. Sex differences were also evident, with women showing lower IC scores and more extensive impairment, consistent with evidence that older women experience greater overall and domain-specific declines, likely reflecting cumulative life-course disadvantages [1,6,10,36,39]. Beyond biological factors, being single and having lower educational attainment were independently associated with worse IC outcomes, underscoring the enduring imprint of social relationships, education, and structural inequalities on trajectories of functional aging [10,15,21,28,36,38,42].

Indicators of economic and social vulnerability were consistently associated with worse IC outcomes. Food insecurity showed consistent associations with lower IC scores and a more extensive impairment across analytical approaches. Although widely associated with disability, poor physical and mental health, and nutritional deficiencies in older adults, its relationship with IC has been less frequently examined [43–46]. Nevertheless, given its potential impact across vitality, cognition, locomotion, and psychological domains, food insecurity may be an important correlate of multidomain IC decline [43–45]. Absence of pension coverage and current non-employment were similarly associated with lower IC and a higher likelihood of more extensive IC impairment across analytical approaches. Pension income has been identified as a structural determinant of healthy aging, particularly in low- and middle-income countries, and is linked to healthier behaviors and greater use of preventive services [38,47,48]. Employment may contribute to preserving IC through sustained social interaction, cognitive engagement, and financial stability, although its effects may vary by occupational conditions, as exit from physically demanding or hazardous occupations may have different implications for health and functional aging [35,36,38,45,48,49]. Lack of healthcare affiliation was also linked to greater IC impairment, consistent with longitudinal evidence showing lower IC among uninsured older adults, independent of other socioeconomic factors, likely reflecting fragmented care and reduced opportunities for early detection and management of factors contributing to IC decline [25,38].

These findings are consistent with the conceptual framework proposed in this study, in which SDH influence IC through both individual social conditions and the accumulation of social disadvantages. The SVI was designed to capture this cumulative dimension of disadvantage using a deficit accumulation approach, integrating multiple social factors that may collectively contribute to lower resilience and greater vulnerability in later life [25,27,50]. In this context, the observed associations between higher social vulnerability and lower IC reinforce the importance of considering broader social and structural conditions when examining healthy aging trajectories.

When socioeconomic disadvantage was examined through a composite social vulnerability score, associations were observed when SVI was categorized into tertiles, whereas significant association was also observed when SVI was modeled as a continuous variable. The strongest inverse associations were observed for locomotion and cognition, followed by vitality and psychological domains, whereas associations with sensory function were comparatively smaller. These findings suggest that social vulnerability disproportionately affects domains linked to physical performance and cognitive reserve, likely reflecting cumulative life-course exposures to adverse working conditions, limited stimulation, nutritional constraints, and restricted opportunities for engagement [26]. In contrast, sensory decline may be more strongly influenced by biological aging and chronic disease processes [6].

Health determinants also played a central role. Chronic disease burden was associated with worse IC outcomes in a graded manner, with multimorbidity conferring the greatest disadvantage. This may reflect a potential bidirectional relationship between multimorbidity and IC, whereby chronic disease accelerates declines in physiological reserve, while reduced IC increases vulnerability to adverse outcomes [13,36,51]. Importantly, despite its close link with chronic disease, IC appears to better predict long-term outcomes than simple disease counts, highlighting the value of a function-centered approach to healthy aging [7,37]. Self-rated health (SRH) was consistently associated with IC. Individuals reporting fair or poor health had lower IC scores and more extensive impairment. Again, this relationship appears bidirectional, as subjective health perceptions both reflect current functional status and predict future IC trajectories [1,10]. While most research has focused on IC decline affecting SRH, emerging evidence indicates that positive SRH is linked to maintaining or improving IC over time [1,5,10]. By capturing a holistic view of physical, mental, and social well-being, SRH may detect early functional decline before it is evident through clinical disease measures alone [29].

Sleep disturbances and falls further reflected advanced functional vulnerability. Sleep disturbances were highly prevalent, particularly among the oldest-old. Evidence suggests that while sleep disturbances may emerge earlier in later life, their impact on IC intensifies from age 75 onward, strongly linking poor or prolonged sleep to declines across multiple IC domains, likely reflecting cumulative aging, pain, multimorbidity, and reduced resilience [52,53]. Falls were described in relation to IC; however, no statistically significant association was observed in adjusted analyses. While prior research has focused on low IC as a predictor of falls, a history of falls also signals multidomain functional decline, highlighting the potentially bidirectional relationship between falls and IC in later life. [10,54].

Finally, smoking was associated with worse IC outcomes. Although no significant association was observed in the ordinal model, smoking was associated with lower IC scores and more extensive impairment in other analytical approaches, suggesting a consistent pattern across models. Current and former smokers had lower IC scores and more extensive impairment, consistent with previous studies reporting an association between smoking and poorer functional aging outcomes [6,13,36].

This study has several strengths. It draws on nationally representative data from the MHAS, enhancing the external validity and generalizability of the findings to community-dwelling older adults in Mexico. The inclusion of adults aged 50 years and older allowed the identification of IC impairment across a broad age spectrum, capturing both early and more advanced stages of functional decline. IC was operationalized using complementary analytical approaches, including domain-specific impairments, the number of affected domains, and a continuous latent IC index derived through factor analysis, reinforcing its multidimensional and dynamic nature. Importantly, beyond examining individual socioeconomic determinants in isolation, we constructed a composite social vulnerability index, enabling the assessment of cumulative disadvantage and its graded associations with IC across domains. This integrative strategy strengthens the robustness of the findings and aligns with the conceptualization of IC as shaped by structural and life-course determinants rather than single exposures.

Several limitations should be acknowledged. The cross-sectional design precludes causal inference and the evaluation of IC trajectories over time; however, it remains appropriate for identifying population-level correlates of IC impairment. Most study variables, including IC domains and health-related factors, were self-reported and may be subject to recall or social desirability bias. In particular, alcohol consumption was assessed only as current use, without information on past consumption, cumulative exposure, quantity, or drinking patterns, which may partly explain the observed associations. Food insecurity was measured using a brief indicator, potentially underestimating gradients of economic vulnerability, and residual confounding by unmeasured life-course and environmental factors cannot be excluded.

Additionally, although the IC construct was developed following the WHO ICOPE framework, its operationalization relied on proxy measures available in the MHAS dataset rather than the original ICOPE screening tool. Therefore, direct comparisons with studies using different instruments or operational definitions of IC should be interpreted with caution and more studies are needed.

## Conclusion

IC impairment is highly prevalent among Mexican adults aged 50 years and older, reflecting substantial multidimensional vulnerability. Our findings reinforce that IC is not driven by aging alone but is closely linked to socioeconomic disadvantage and health-related conditions. In this nationally representative sample, low educational attainment, economic insecurity, limited access to healthcare, multimorbidity, poor self-rated health, sleep disturbances, and adverse lifestyle factors were consistently associated with more extensive IC impairment. Notably, when examined through a composite social vulnerability index, clear graded associations emerged across IC domains, underscoring the cumulative impact of structural disadvantage on functional reserve.

Together, these results underscore the value of IC as an integrative, function-centered framework for characterizing vulnerability beyond traditional disease-based approaches. Strengthening strategies that address social vulnerability and modifiable health factors may be essential for preserving functional ability and promoting healthy aging at the population level.

## Supporting information

S1 Table Operationalization of Intrinsic Capacity Domains Using MHAS 2021 Variables. (DOCX)

S1 Fig Standardized Factor Loadings for the Intrinsic Capacity Measurement Model. (TIFF)

S2 Fig Conceptual framework linking social determinants of health (SDH), social vulnerability, the Social Vulnerability Index (SVI), and intrinsic capacity. (TIFF)

S2 Table Sensitivity analysis excluding employment status from the Social Vulnerability Index. (DOCX)

S3 Table Variance Inflation Factors (VIFs) for covariates included in multivariable regression models. (DOCX)

## References

[1] Bernal MC, Batista E, Martínez-Ballesté A, Solanas A. A functional approach to model intrinsic capacity in ageing trajectories. Sci Rep. 2025;15(1):7878. doi: 10.1038/s41598-025-92271-7 40050411 PMC11885805

[2] Sánchez-Sánchez JL, Lu W-H, Gallardo-Gómez D, Del Pozo Cruz B, de Souto Barreto P, Lucia A, et al. Association of intrinsic capacity with functional decline and mortality in older adults: a systematic review and meta-analysis of longitudinal studies. Lancet Healthy Longev. 2024;5(7):e480–92. doi: 10.1016/S2666-7568(24)00092-8 38945130

[3] Beard JR, Officer AM, Cassels AK. The world report on ageing and health. Gerontologist. 2016. doi: 10.1093/geront/gnw037 26994257

[4] Lu W-H, Guyonnet S, Martinez LO, Lucas A, Parini A, Vellas B, et al. Association between aging-related biomarkers and longitudinal trajectories of intrinsic capacity in older adults. Geroscience. 2023;45(6):3409–18. doi: 10.1007/s11357-023-00906-2 37620614 PMC10643641

[5] Wang Y, Tang N, Shao M, Song J, Su Q, Gao Y. Transitions and trajectories in intrinsic capacity states over time: a systematic review. BMC Geriatr. 2025;25(1):407. doi: 10.1186/s12877-025-05938-1 40468176 PMC12135290

[6] Fuentealba M, Rouch L, Guyonnet S, Lemaitre J-M, de Souto Barreto P, Vellas B, et al. A blood-based epigenetic clock for intrinsic capacity predicts mortality and is associated with clinical, immunological and lifestyle factors. Nat Aging. 2025;5(7):1207–16. doi: 10.1038/s43587-025-00883-5 40467932 PMC12270914

[7] Lee W-J, Peng L-N, Lin M-H, Loh C-H, Hsiao F-Y, Chen L-K. Intrinsic capacity differs from functional ability in predicting 10-year mortality and biological features in healthy aging: results from the I-Lan longitudinal aging study. Aging (Albany NY). 2023;15(3):748–64. doi: 10.18632/aging.204508 36750172 PMC9970311

[8] Tay L, Tay E-L, Mah SM, Latib A, Koh C, Ng Y-S. Association of Intrinsic Capacity with Frailty, Physical Fitness and Adverse Health Outcomes in Community-Dwelling Older Adults. J Frailty Aging. 2023;12(1):7–15. doi: 10.14283/jfa.2022.28 36629078 PMC8966852

[9] Zhang N, Zhang H, Sun M-Z, Zhu Y-S, Shi G-P, Wang Z-D, et al. Intrinsic capacity and 5-year late-life functional ability trajectories of Chinese older population using ICOPE tool: the Rugao Longevity and Ageing Study. Aging Clin Exp Res. 2023;35(10):2061–8. doi: 10.1007/s40520-023-02489-6 37460764

[10] Shen S, Xie Y, Zeng X, Chen L, Guan H, Yang Y, et al. Associations of intrinsic capacity, fall risk and frailty in old inpatients. Front Public Health. 2023;11:1177812. doi: 10.3389/fpubh.2023.1177812 37886051 PMC10598390

[11] Cesari M, Araujo de Carvalho I, Amuthavalli Thiyagarajan J, Cooper C, Martin FC, Reginster J-Y, et al. Evidence for the Domains Supporting the Construct of Intrinsic Capacity. J Gerontol A Biol Sci Med Sci. 2018;73(12):1653–60. doi: 10.1093/gerona/gly011 29408961

[12] Beard JR, Chen M. Intrinsic Capacity as a Composite Outcome Measure: Opportunities and Challenges. J Nutr Health Aging. 2023;27(6):398–400. doi: 10.1007/s12603-023-1923-z 37357320 PMC12880024

[13] Integrated care for older people (ICOPE): guidance for person-centred assessment and pathways in primary care. Geneva: World Health Organization. https://www.who.int/publications/i/item/9789240103726

[14] Cao X, Yi X, Chen H, Tian Y, Li S, Zhou J. Prevalence of intrinsic capacity decline among community-dwelling older adults: a systematic review and meta-analysis. Aging Clin Exp Res. 2024;36(1):157. doi: 10.1007/s40520-024-02816-5 39088112 PMC11294388

[15] Jiang X, Chen F, Yang X, Yang M, Zhang X, Ma X, et al. Effects of personal and health characteristics on the intrinsic capacity of older adults in the community: a cross-sectional study using the healthy aging framework. BMC Geriatr. 2023;23(1):643. doi: 10.1186/s12877-023-04362-7 37817083 PMC10566030

[16] Bencivenga L, Strumia M, Rolland Y, Guyonnet S, Parini A, Cestac P, et al. Visit-to-visit blood pressure variability is associated with intrinsic capacity decline: Results from the MAPT Study. Eur J Intern Med. 2024;125:82–8. doi: 10.1016/j.ejim.2024.03.001 38499456

[17] Xu L, Raval M. Intergenerational reminiscence program in improving generation connections: a radar analysis. Innovation in Aging. 2023;7(Supplement_1):893–893. doi: 10.1093/geroni/igad104.2874

[18] Gutiérrez-Robledo LM, García-Chanes RE, Pérez-Zepeda MU. Screening intrinsic capacity and its epidemiological characterization: A secondary analysis of the Mexican Health and Aging Study. Revista Panamericana de Salud Publica/Pan American Journal of Public Health. 2021;45. doi: 10.26633/RPSP.2021.121 PMC8437155 34531905

[19] Shin JH, Sagong H, Yoon JY. The relationship between trajectories of intrinsic capacity and differences in the risk of functional ability decline in community-dwelling older adults: a socio-ecological approach. Arch Gerontol Geriatr. 2025;131:105772. doi: 10.1016/j.archger.2025.105772 39884088

[20] Abud T, Kounidas G, Martin KR, Werth M, Cooper K, Myint PK. Determinants of healthy ageing: a systematic review of contemporary literature. Aging Clin Exp Res. 2022;34(6):1215–23. doi: 10.1007/s40520-021-02049-w 35132578 PMC8821855

[21] Salinas-Rodríguez A, Fernández-Niño JA, Rivera-Almaraz A, Manrique-Espinoza B. Intrinsic capacity trajectories and socioeconomic inequalities in health: the contributions of wealth, education, gender, and ethnicity. Int J Equity Health. 2024;23(1):48. doi: 10.1186/s12939-024-02136-0 38462637 PMC10926672

[22] Kemoun P, Ader I, Planat-Benard V, Dray C, Fazilleau N, Monsarrat P, et al. A gerophysiology perspective on healthy ageing. Ageing Res Rev. 2022;73:101537. doi: 10.1016/j.arr.2021.101537 34883201

[23] George PP, Lun P, Ong SP, Lim WS. A Rapid Review of the Measurement of Intrinsic Capacity in Older Adults. J Nutr Health Aging. 2021;25(6):774–82. doi: 10.1007/s12603-021-1622-6 34179933 PMC7966899

[24] Naidoo M, Shephard W, Kambewe I, Mtshali N, Cope S, Rubio FA, et al. Incorporating social vulnerability in infectious disease mathematical modelling: a scoping review. BMC Med. 2024;22(1):125. doi: 10.1186/s12916-024-03333-y 38500147 PMC10949739

[25] Zhao B, Liu Z, Fu Y, Zhang H, Wu J, Lai C, et al. Social Determinants of Intrinsic Capacity: A National Cohort Study. Am J Prev Med. 2024;66(3):559–67. doi: 10.1016/j.amepre.2023.10.008 37844711

[26] Marmot M, Allen J, Bell R, Bloomer E, Goldblatt P, Consortium for the European Review of Social Determinants of Health and the Health Divide. WHO European review of social determinants of health and the health divide. Lancet. 2012;380(9846):1011–29. doi: 10.1016/S0140-6736(12)61228-8 22964159

[27] Mah JC, Penwarden JL, Pott H, Theou O, Andrew MK. Social vulnerability indices: a scoping review. BMC Public Health. 2023;23(1):1253. doi: 10.1186/s12889-023-16097-6 37380956 PMC10304642

[28] Si Y, Hanewald K, Chen S, Li B, Bateman H, Beard JR. Life-course inequalities in intrinsic capacity and healthy ageing, China. Bull World Health Organ. 2023;101(5):307-316C. doi: 10.2471/BLT.22.288888 37131938 PMC10140694

[29] Chu S-P, Wu C-Y, Lai H-Y, Chen I-T, Meng L-C, Liang C-K, et al. Age-specific associations between intrinsic capacity impairments and self-rated health in community-dwelling adults: Insights from Taiwan longitudinal study on aging. J Nutr Health Aging. 2025;29(11):100688. doi: 10.1016/j.jnha.2025.100688 41033191 PMC12513283

[30] Zheng T, Chenyang L, Shangjun L, Shuqin X, Jiaqi D, Yanyan Z, et al. Measuring and decomposing inequalities in intrinsic capacity among older adults in china: from an urban-rural divide perspective. Aging Clin Exp Res. 2025;38(1):33. doi: 10.1007/s40520-025-03284-1 41460460 PMC12775099

[31] Beard JR, Jotheeswaran AT, Cesari M, Araujo de Carvalho I. The structure and predictive value of intrinsic capacity in a longitudinal study of ageing. BMJ Open. 2019;9(11):e026119. doi: 10.1136/bmjopen-2018-026119 31678933 PMC6830681

[32] Beard JR, Si Y, Liu Z, Chenoweth L, Hanewald K. Intrinsic Capacity: Validation of a New WHO Concept for Healthy Aging in a Longitudinal Chinese Study. J Gerontol A Biol Sci Med Sci. 2022;77(1):94–100. doi: 10.1093/gerona/glab226 34343305

[33] Aliberti MJR, Bertola L, Szlejf C, Oliveira D, Piovezan RD, Cesari M, et al. Validating intrinsic capacity to measure healthy aging in an upper middle-income country: Findings from the ELSI-Brazil. Lancet Reg Health Am. 2022;12:100284. doi: 10.1016/j.lana.2022.100284 36776430 PMC9903598

[34] Aguilar-Navarro SG, Fuentes-Cantú A, Ávila-Funes JA, García-Mayo EJ. Validez y confiabilidad del cuestionario del ENASEM para la depresión en adultos mayores. Salud pública Méx. 2007;49(4):256–62. doi: 10.1590/s0036-36342007000400005 17710274

[35] Zhao Y, Chen Y, Xiao LD, Liu Q, Nan J, Li X, et al. Intrinsic capacity trajectories, predictors and associations with care dependence in community-dwelling older adults: A social determinant of health perspective. Geriatr Nurs. 2024;56:46–54. doi: 10.1016/j.gerinurse.2023.12.022 38237340

[36] Tang L, Binti Rasudin NS, Dong Y, Yusuf A. Determinants of intrinsic capacity among older adults in low- and middle-income countries: A scoping review. Belitung Nurs J. 2025;11(6):661–73. doi: 10.33546/bnj.4007 41312041 PMC12648234

[37] Ramírez-Vélez R, Iriarte-Fernández M, Santafé G, Malanda A, Beard JR, Garcia-Hermoso A, et al. Association of intrinsic capacity with incidence and mortality of cardiovascular disease: Prospective study in UK Biobank. J Cachexia Sarcopenia Muscle. 2023;14(5):2054–63. doi: 10.1002/jcsm.13283 37434422 PMC10570093

[38] Huang Z-T, Lai ETC, Luo Y, Woo J. Social determinants of intrinsic capacity: A systematic review of observational studies. Ageing Res Rev. 2024;95:102239. doi: 10.1016/j.arr.2024.102239 38382677

[39] Meng LC, Chuang HM, Lu WH, Lee WJ, Liang CK, Loh CH, et al. Multi-Trajectories of Intrinsic Capacity Decline and Their Impact on Age-Related Outcomes: A 20-Year National Longitudinal Cohort Study. Aging Dis. 2023;15:2697. doi: 10.14336/AD.2023.1115-1 38029399 PMC11567246

[40] Liao X, Shen J, Li M. Effects of multi-domain intervention on intrinsic capacity in older adults: A systematic review of randomized controlled trials (RCTs). Exp Gerontol. 2023;174:112112. doi: 10.1016/j.exger.2023.112112 36736466

[41] Zhu L-Y, Chan R, Kwok T, Cheng KC-C, Ha A, Woo J. Effects of exercise and nutrition supplementation in community-dwelling older Chinese people with sarcopenia: a randomized controlled trial. Age Ageing. 2019;48(2):220–8. doi: 10.1093/ageing/afy179 30462162

[42] Hui H. The Influence Mechanism of Education on Health from the Sustainable Development Perspective. J Environ Public Health. 2022;2022:7134981. doi: 10.1155/2022/7134981 35910750 PMC9328960

[43] Aljahdali AA, Na M, Leung CW. Food insecurity and health-related quality of life among a nationally representative sample of older adults: cross-sectional analysis. BMC Geriatr. 2024;24(1):126. doi: 10.1186/s12877-024-04716-9 38302907 PMC10835917

[44] Na M, Dou N, Ji N, Xie D, Huang J, Tucker KL, et al. Food Insecurity and Cognitive Function in Middle to Older Adulthood: A Systematic Review. Adv Nutr. 2020;11(3):667–76. doi: 10.1093/advances/nmz122 31711095 PMC7231583

[45] Aljahdali AA, Ludwig-Borycz E, Leung CW. Food insecurity, inflammation, and immune function among older US adults: Findings from the health and Retirement study. Brain Behav Immun. 2024;119:28–35. doi: 10.1016/j.bbi.2024.03.034 38552920 PMC11162895

[46] Hill CM, Tseng AS, Holzhauer K, Littman AJ, Jones-Smith JC. Association between health care access and food insecurity among lower-income older adults with multiple chronic conditions in Washington State, USA. Public Health Nutr. 2023;26(1):199–207. doi: 10.1017/S1368980022001240 35603699 PMC11077446

[47] Yang D, Ren Z, Zheng G. The impact of pension insurance types on the health of older adults in China: a study based on the 2018 CHARLS data. Front Public Health. 2023;11:1180024. doi: 10.3389/fpubh.2023.1180024 37333531 PMC10272461

[48] Zhu AYF, Chou KL. Health Outcomes of Social Pension Expansion: A Quasi-Experiment Among Older Adults in Hong Kong. J Appl Gerontol. 2024;43(1):26–36. doi: 10.1177/07334648231195493 37614125

[49] Kuhn A. The complex effects of retirement on health. izawol. 2018. doi: 10.15185/izawol.430

[50] Andrew MK, Mitnitski AB, Rockwood K. Social vulnerability, frailty and mortality in elderly people. PLoS One. 2008;3(5):e2232. doi: 10.1371/journal.pone.0002232 18493324 PMC2375054

[51] Zhang M, Xu Y, Xing Y, Li H. Association between multimorbidity and intrinsic capacity among older Chinese adults: evidence from the CHARLS 2011-2015. Eur Geriatr Med. 2025;16(4):1207–16. doi: 10.1007/s41999-025-01232-w 40418266

[52] Yuan Q, Yue X, Wang M, Yang F, Fu M, Liu M, et al. Association between pain, sleep and intrinsic capacity in Chinese older adults: Evidence from CHARLS. J Nutr Health Aging. 2025;29(3):100466. doi: 10.1016/j.jnha.2024.100466 39742576 PMC12180043

[53] Zhou B, Ma R, Wang M, Wang Y. Dose-response relationship between nighttime sleep duration and intrinsic capacity declines among Chinese elderly: a cross-sectional study from CHARLS. BMC Public Health. 2025;25(1):1034. doi: 10.1186/s12889-025-22294-2 40098027 PMC11917026

[54] Liu A, You Y, Wang Y, Li L, Yuan J. Research on intrinsic capacity as a predictor of falls and disability in community-dwelling elderly. Frontiers in Aging. 2025;6. doi: 10.3389/fragi.2025.1589369 PMC12162613 40519669

