# Supplementary material for: Social vulnerability and health determinants of Intrinsic Capacity in Mexican older adults: Evidence from the Mexican Health and Aging Study round 2021
Source: PLoS One. 2026 Aug 7;21(8):e0353903. doi: 10.1371/journal.pone.0353903 (PMC13450843; doi:10.1371/journal.pone.0353903)
Supplement: Supplementary file 1 [file pone.0353903.s008.pdf]

Mexico City June 11 2026

Manuscript Title: Social Vulnerability and Health Determinants of Intrinsic Capacity in Mexican Older Adults: Evidence from the Mexican Health and Aging Study round 2021.

Manuscript ID: PONE-D-26-15851

Dear Reviewers,

We sincerely thank you for your thorough evaluation of our manuscript and for the constructive comments and suggestions. We have carefully revised the manuscript accordingly. Below, we provide a detailed, point-by-point response to each comment. All changes have been incorporated into the revised manuscript and are highlighted using track changes.

Comments from Reviewer #1

1. **Comment 1:**The methodology is adequate for a cross-sectional, hypothesis-generating study but has significant flaws that limit causal inference and internal validity for certain domains (notably locomotion and SVI). The authors have done a commendable job operationalizing IC using CFA and constructing a cumulative SVI. However, the extremely high prevalence of locomotor impairment (96.8%) threatens domain discrimination, and the contradictory findings for the SVI (continuous vs. categorical) and alcohol consumption raise concerns about model specification and residual confounding.
    a. **Response:** We thank the reviewer for these valuable comments.

       Regarding the locomotion domain, we re-examined its operationalization and found that the household maintenance item was highly prevalent and contributed substantially to the very high prevalence of locomotor impairment. Additionally, this indicator showed the weakest contribution in the CFA. Therefore, we revised the locomotion domain by excluding this item and retaining measures more directly related to physical mobility (walking several blocks, climbing stairs, and rising from a chair).

       Regarding the SVI, we acknowledge the different findings obtained when modeling the index as a continuous versus categorical variable. We believe this reflects a potential non-linear association between social vulnerability and intrinsic capacity. We have clarified this interpretation and acknowledged it as a study limitation.

       Regarding alcohol consumption, the MHAS only captures current alcohol use and does not provide information on quantity, frequency, lifetime exposure, or former drinking status. Therefore, residual confounding and a potential

"healthy drinker" effect cannot be excluded. We have expanded the limitations section accordingly.

Finally, we further emphasize that the cross-sectional design precludes causal inference and that the findings should be interpreted as associations useful for hypothesis generation.

2. **Comment 2:**
    a. Construction issues:
        i. SVI includes "living alone." Living alone is not universally a deficit in older adults, as it may indicate independence and resources. Social isolation would be a better choice.
        ii. SVI includes "not currently employed" but for adults ≥70 years, this is normative, not a social vulnerability deficit.
    b. **Response:** We thank the reviewer for this comment. We agree that living alone does not universally represent a deficit and may, in some cases, reflect autonomy and independence. However, evidence from both Mexican and international studies has linked living alone to adverse outcomes in older adults. In Mexico, Franco-Alvarez et al. (2007) identified living alone as a determinant of malnutrition risk among community-dwelling older adults, and Rodríguez-Tadeo et al. (2012) reported that living alone remained independently associated with nutritional risk after multivariable adjustment.Internationally, Abell and Steptoe (2021) found that living alone was associated with a 20% higher mortality risk among older adults. Furthermore, living alone was explicitly included as a component of the Social Vulnerability Index proposed by Andrew, Mitnitski, and Rockwood (2008). Therefore, although living alone should not be interpreted as a vulnerability deficit per se, we considered it a meaningful indicator of potential social vulnerability and retained it as one component of the multidimensional SVI.

    Regarding employment status, we agree that retirement and labor-force exit are common transitions in later life and may not uniformly reflect vulnerability across all older adults. To address this concern, we conducted a sensitivity analysis excluding employment status from the SVI and repeated all SVI analyses. The association between social vulnerability and intrinsic capacity remained significant. Compared with the lowest vulnerability tertile, intermediate vulnerability was associated with lower intrinsic capacity (β = −0.202; 95% CI: −0.237 to −0.168; $p < 0.001$), and high vulnerability showed an even stronger association (β = −0.377; 95% CI: −0.420 to −0.334; $p < 0.001$). Similarly, the continuous SVI excluding employment status remained significantly associated with lower intrinsic capacity (β = −0.154; 95% CI:

−0.168 to −0.140; p < 0.001). These results are presented in Supplementary Table S2 and indicate that the association between social vulnerability and intrinsic capacity was not driven by the inclusion of employment status in the index.

3. **Comment 3:**
   The manuscript uses potentially causal language (e.g., "identify early vulnerability," "IC predicts," "social disadvantage shapes IC") despite the cross-sectional design, which does not allow for establishing temporality.
    a. **Response:** We thank the reviewer for this important observation. We agree that the cross-sectional design does not allow the establishment of temporality or causal inference. Accordingly, we carefully reviewed the manuscript and revised statements that could be interpreted as causal. Terms such as "predict," "shape," "contribute to," and "identify early vulnerability" were replaced with more appropriate language emphasizing associations, correlates, or relationships. We have also reinforced in the Discussion and Conclusion sections that the findings should be interpreted as cross-sectional associations rather than causal effects.These changes have been incorporated in lines 69, 70, 89, 90, 120, 521 of the revised manuscript.

4. **Comment 4:**
   The Discussion includes causal interpretations (e.g., "smoking's role in accelerating functional aging") that are not supported by the study design.
    a. **Response:** We thank the reviewer for this observation. We agree that the cross-sectional design does not support causal interpretations regarding the effects of smoking or other factors on intrinsic capacity. Accordingly, we revised the Discussion to avoid causal language and to emphasize associations. Specifically, statements referring to smoking as "accelerating functional aging" were rephrased to indicate that smoking was associated with lower intrinsic capacity and more extensive impairment in our study population. Similar revisions were made where necessary to ensure that all findings are interpreted as associations rather than causal effects. These changes have been incorporated in lines 478-479 of the revised manuscript.

5. **Comment 9:**
   Operationalization of IC Domains – Potential Misalignment with WHO ICOPE
    a. While the authors state they follow the WHO ICOPE framework, several domain proxies are questionable, in particular the locomotor domain: Difficulty with 4 activities (walking blocks, stairs, home maintenance, rising from chair) is overly sensitive: 96.8% prevalence of impairment. This is likely ceiling

effect, rendering the domain almost useless for discriminating among community-dwelling adults. “Home maintenance/repairs” is heavily influenced by gender roles and housing conditions, not pure locomotion.

b. **Response:** We appreciate the reviewer’s observation. We agreed that the original operationalization of the locomotion domain showed limited discriminatory capacity, as the prevalence of impairment reached 96.8%. After further evaluation, we identified the household maintenance/repair item as the primary contributor to this issue, as it may reflect household roles and environmental factors in addition to mobility limitations. Accordingly, we excluded this item and redefined the locomotion domain using indicators more directly related to mobility (walking several blocks, climbing stairs, and rising from a chair). This modification reduced the prevalence of locomotor impairment from 96.8% to 43.1%, substantially improving domain discrimination. All analyses were repeated using the revised definition, and the main findings remained materially unchanged, supporting the robustness of the results. The revised operationalization has been updated throughout the manuscript.

Comments from Reviewer #2

Introduction

1. **Comment 1:** The authors should include a clear conceptual definition of social vulnerability, as well as explain how this concept is linked to the social determinants of health (SDH) framework.
    a. **Response**: We thank the reviewer for this valuable observation. In response, we added a clear conceptual definition of social vulnerability and further clarified its relationship with the Social Determinants of Health (SDH) framework. These additions have been incorporated in lines 115–124 of the revised manuscript.

2. **Comment 2:** Additionally, the information presented in lines 122-124 is unclear and should be rewritten to improve clarity.
    a. Response: We thank the reviewer for this comment. The text has been revised to improve clarity and readability, and we hope the revised version provides a clearer presentation of the intended concept. These changes have been incorporated in lines 130–136 of the revised manuscript.

3. **Comment 3:** The research objective is not clearly stated. Considering the design of the MHAS and the use of only the 2021 wave, the term “evaluate” is not appropriate. Furthermore, the expressions “key SDH” and “graded association” require clearer definitions.

    a. **Response:** We appreciate this observation. To improve clarity, we revised the study objective to better align with the cross-sectional design, clarified the social determinants of health considered in the analyses, and more clearly described the progressive association observed between higher levels of social vulnerability and lower intrinsic capacity. These changes have been incorporated in lines 141, 317 y 372 of the revised manuscript.

4. **Comment 4:** The introduction is framed using a longitudinal perspective; however, the analysis relies on a single wave of the MHAS. This creates an inconsistency between the conceptual and statistical approaches.
    a. Response: We thank the reviewer for this important observation. We agree that the previous version of the introduction contained language that could imply a longitudinal perspective and was not fully aligned with the cross-sectional nature of our analysis based on a single wave of the MHAS. In response, we revised the introduction, including the wording and supporting evidence, to adopt a more cross-sectional framework and avoid language suggestive of temporal trajectories or causal pathways.

5. **Comment 5:** Moreover, the main focus of the study is the analysis of SDH associated with IC, which implies statistical associations rather than measures of inequality. Nonetheless, several parts of the introduction refer to inequalities in IC related to the differential impact of SDH. This inequality-based perspective does not contribute clearly to the stated objective and should either be properly justified or removed.
    a. **Response:** the change was done

Methodology

1. **Comment 9:**
   The information in lines 152–155 regarding the MHAS 2024 wave is unnecessary and should be removed.
    a. **Response:** We appreciate the reviewer's observation. The statement referring to the 2024 MHAS wave was removed, as it was not essential to the description of the study design or data source. The Methods section has been revised accordingly.

2. **Comment 10:**
   Given the importance of constructing the IC index using MHAS data, a supplementary file detailing all questions, variables, and procedures used to generate the index should be provided to improve transparency and reproducibility.
   a. **Response:** Thank you for this comment. We agree that detailed documentation of the IC index construction is essential for transparency and reproducibility. Accordingly, we have added a supplementary table describing the MHAS 2021 variables, operationalization procedures, and coding rules used to derive each IC domain and the overall IC index.

3. **Comment 11:**
   There is a conceptual issue that may also lead to statistical problems in the construction of the social vulnerability index, as it includes variables that are also used as covariates (e.g., education, employment status, healthcare affiliation), which may introduce multicollinearity in the regression models.
   a. **Response:** We appreciate the reviewer's observation. We would like to clarify that the individual social variables included in the Social Vulnerability Index (SVI) were not entered simultaneously with the SVI in the same regression models. Tables 2–4 examined the associations of individual social and health-related factors with intrinsic capacity, whereas Table 5 evaluated the association between the composite SVI and intrinsic capacity. Therefore, the SVI components and the SVI itself were not modeled concurrently, minimizing concerns regarding multicollinearity.

      To further address this concern, we assessed multicollinearity among covariates included in the regression models using variance inflation factors (VIFs). All VIF values were low (range: 1.04–1.78; mean VIF = 1.26), indicating that multicollinearity was not a concern (Supplementary Table S3).

      In addition, we conducted a sensitivity analysis excluding employment status from the SVI and repeated all SVI analyses. The results remained materially unchanged (Supplementary Table S2), supporting the robustness of the findings and indicating that the observed associations were not driven by overlap among individual SVI components.

4. **Comment 12:**
   The inclusion of this index should be reconsidered, especially since the concept of social vulnerability is not clearly defined in the introduction nor properly integrated into the SDH framework throughout the manuscript.
   a. Response: We appreciate the reviewer's observation. In response, we strengthened the conceptual framework presented in the Introduction by incorporating explicit definitions of both Social Determinants of Health (SDH) and social vulnerability, and by clarifying the relationship between these concepts. We also expanded the rationale for including the Social Vulnerability Index (SVI) by incorporating recent literature on social

vulnerability indices and the social determinants of intrinsic capacity (Mah et al., 2023; Zhao et al., 2024). These revisions were incorporated into the Introduction (approximately lines 115–130 of the revised manuscript).

To further improve clarity, we developed a conceptual framework illustrating the relationship between SDH, social vulnerability, and intrinsic capacity (Supplementary Figure S1). This figure highlights how individual social determinants contribute to cumulative social vulnerability, which in turn is associated with intrinsic capacity outcomes. Together, these revisions provide a clearer theoretical justification for the inclusion of the SVI in the present study.

5. **Comment 13:**
The regression models lack coherence. The use of odds ratios and coefficients, along with different categorizations of variables, creates confusion regarding the main analytical approach.
    a. **Response:** We appreciate the reviewer's observation. To improve clarity and coherence, we revised the Statistical Analysis section (lines 259–282) to more clearly describe the rationale for each analytical approach. Specifically, we clarified that ordinal logistic regression was used for the ordered IC impairment groups, linear regression for the continuous latent IC score, multinomial logistic regression for IC tertiles, and linear regression models for the association between the Social Vulnerability Index (SVI) and the continuous IC score. We also explicitly stated the corresponding effect measures reported for each model (ORs, RRRs, and β coefficients). These revisions were intended to clarify that the different models reflect complementary operationalizations of intrinsic capacity rather than different analytical objectives.

6. **Comment 14:**
A stronger justification is needed for using multiple modeling strategies beyond simple comparison. Selecting a single, well-justified model would improve clarity and focus.
    a. **Response:** We appreciate the reviewer's observation. Our intention was not to compare competing modeling strategies, but rather to examine IC using complementary operationalizations that capture different aspects of the construct. Because IC can be represented as the number of impaired domains, a continuous latent score derived from factor analysis, or clinically interpretable categories, different regression models were required according to the scale of the outcome variable.

    To improve clarity, we revised the Statistical Analysis section (lines 259–282) to explicitly describe the rationale for each modeling approach. Importantly,

the findings were highly consistent across models, with similar patterns observed regardless of whether IC was analyzed as ordered impairment groups, a continuous latent score, or tertiles of IC. We believe that this complementary approach strengthens the robustness and interpretability of the findings rather than representing alternative analytical strategies.

Results

1. **Comment 15:**
   The results should be revised in light of the methodological concerns mentioned above.
   a. **Response:** We appreciate the reviewer's observation. Following the methodological revisions implemented in response to the comments above, we re-ran all descriptive and multivariable analyses and updated the corresponding tables, figures, and results throughout the manuscript. In particular, the locomotion domain was redefined to improve discriminatory capacity, the Social Vulnerability Index framework was further clarified, and additional sensitivity and multicollinearity analyses were conducted (Supplementary Tables S2 and S3). Although some estimates changed slightly, the overall direction and interpretation of the findings remained consistent, supporting the robustness of the study conclusions.

2. **Comment 16:**
   There is an inconsistency between the sample size reported in the text and in Figure 1, which should be carefully verified.
   a. **Response:** Thank you for highlighting this discrepancy. We carefully reviewed the sample selection process and verified the reported numbers. The inconsistency between the text and Figure 1 has been corrected, and all sample sizes were rechecked to ensure consistency throughout the manuscript.

3. **Comment 17:**
   Since sample weights were applied, weighted (expanded) estimates should be reported throughout the results.
   a. **Response:** We appreciate the reviewer's observation. All descriptive and multivariable analyses were conducted using the complex survey design of MHAS through the `svy` procedures in Stata, incorporating sampling weights, clustering, and stratification. We revised the manuscript to clarify that percentages, prevalence estimates, and regression results reflect weighted estimates representative of the Mexican population aged 50 years and older, whereas the reported sample size corresponds to the unweighted analytic sample (N = 13,403).

4. **Comment 18:**
   The inclusion of multiple regression models (odds ratios vs. coefficients, different categorizations) unnecessarily lengthens the manuscript without adding substantial value, given the similarity of the findings. The analysis should either be simplified or more strongly justified.
   a. **Response:** We recognize the reviewer's concern regarding the use of multiple analytical approaches. Our intention was not to present alternative analytical strategies, but rather to evaluate intrinsic capacity using complementary operationalizations that capture different aspects of the construct. Specifically, IC was examined as the number of impaired domains, a continuous latent score derived from factor analysis, and tertiles of the continuous score. Each approach required a different modeling strategy according to the scale of the outcome variable.

      To improve clarity, we strengthened the justification for these analyses in the Statistical Analysis section (lines 259–282) and clarified the rationale for each model. Importantly, the consistency of findings across models supports the robustness of the observed associations and provides convergent evidence regarding the factors associated with intrinsic capacity.

5. **Comment 19:**
   Post-estimation diagnostics, such as VIF, should be reported.
   a. Response: We thank the reviewer for this suggestion. Post-estimation diagnostics were conducted to assess potential multicollinearity among covariates. Variance inflation factors (VIFs) were low for all variables (range: 1.04–1.78; mean VIF = 1.26), indicating that multicollinearity was not a concern. These results have been added as Supplementary Table S3.

6. **Comment 20:**
   Supplementary Figure 1 is neither clearly presented nor referenced in the manuscript.
   a. **Response:** the change was done un the new figure 1

Discussion

1. **Comment 21:**
   The discussion largely repeats the results using different wording and provides general statements without deeper analytical development; a more critical and in-depth discussion is needed.

a. **Response:** The discussion has been reviewed in detail, taking into account your observations.

2. **Comment 22:**
   The discussion should explicitly address the implications of the findings for clinical practice and public policy, as well as the main contributions of the study.
   a. **Response:** yes, we has been reviewed in detail, taking into account your observations.

3. **Comment 23:**
   There is a lack of consistency between the conceptual framework presented in the introduction and the discussion of results. Concepts such as SDH and social vulnerability are not adequately revisited or interpreted.
   a. **Response:** In response, we revised the Discussion to strengthen the connection between the conceptual framework presented in the Introduction and the interpretation of the findings. Specifically, we expanded the discussion of Social Determinants of Health (SDH) and social vulnerability, emphasizing how individual social factors and the cumulative Social Vulnerability Index (SVI) reflect broader social and structural conditions associated with intrinsic capacity. We also incorporated a new paragraph in the Discussion (lines 441–449) explicitly linking the observed associations to the SDH and social vulnerability framework. In addition, we developed a conceptual framework (Supplementary Figure S1) illustrating the relationship between SDH, social vulnerability, and intrinsic capacity. These revisions improve the integration of the conceptual framework throughout the manuscript.

4. **Comment 24:**
   It should be acknowledged as a limitation that, although the MHAS allows for longitudinal analysis, this study is restricted to the 2021 wave, and a justification for this choice should be provided.
   a. **Response:** thank you for the recommendation; we are analyzing a longitudinal secondary analysis that would reinforce the construct Intrinsic Capacity

5. **Comment 25:**
   Another limitation that should be explicitly stated is that the IC index constructed in this study is not directly comparable with those used in other research.
   a. **Response:** We appreciate the reviewer’s observation. We agree that direct comparisons of intrinsic capacity (IC) across studies should be made with caution, as operational definitions may vary depending on the available data

sources and measurement instruments. In the present study, IC was constructed using proxy measures available in the Mexican Health and Aging Study (MHAS), following the WHO ICOPE framework and approaches used in previous population-based studies. To improve transparency and facilitate interpretation, we added Supplementary Table S1, which details the operationalization of each IC domain and its correspondence with the WHO ICOPE framework. We also added a statement in the Limitations section (lines 520–524) acknowledging that differences in measurement instruments and operational definitions may limit direct comparability with IC indices reported in other studies.

6. **Comment 26:**
   The content in lines 496–500 is repetitive and should be revised or removed.
   a. **Response:** the change was done thanks

We appreciate the time and effort invested by the Academic Editor and reviewers in evaluating our work. We have carefully considered all comments and revised the manuscript accordingly. We hope that the revisions adequately address all concerns raised. We remain at your disposal for any further clarification and look forward to your response.

Sincerely,

Sara G. Aguilar Navarro

Dr. Sara G. Aguilar Navarro

On behalf of all authors
