# Supplementary material for: Social vulnerability and health determinants of Intrinsic Capacity in Mexican older adults: Evidence from the Mexican Health and Aging Study round 2021
Source: PLoS One. 2026 Aug 7;21(8):e0353903. doi: 10.1371/journal.pone.0353903 (PMC13450843; doi:10.1371/journal.pone.0353903)
Supplement: Supplementary file 2 [file pone.0353903.s001.docx]

**S1 Table.** Operationalization of Intrinsic Capacity Domains Using MHAS 2021 Variables

| **ICOPE Domain** | **MHAS 2021 Variable and Operationalization** | **Scoring** |
| --- | --- | --- |
| **Cognition** | **E.11 Temporal orientation.**  Participants were asked to report the current day, month, and year. Impairment was defined as ≥1 incorrect response. | 1 = Impaired if at least one response was incorrect  0 = Not impaired |
|  | **E.14 Immediate verbal recall.** Assessed using the immediate 8-word recall test. Impairment was defined as recall of <3 words. | 1 = Impaired if fewer than 3 words were recalled  0 = Not impaired |
|  | **Composite cognitive impairment.** Temporal orientation and verbal recall were combined into a binary indicator. | 1 = Impairment in either component  0 = No impairment |
| **Psychological** | **C.49 Depressive symptoms.**  Assessed using a modified nine-item version of the Center for Epidemiologic Studies Depression Scale (CES-D), which evaluates depressive symptoms experienced during the previous week, including depressed mood, effort, restless sleep, happiness, loneliness, enjoyment of life, sadness, fatigue, and energy level.  Depressive symptoms were defined as a score ≥5. | 1 = Depressive symptoms (CES-D score ≥5)  0 = No depressive symptoms (CES-D score <5) |
| **Sensory** | **C.42 Vision.**  Self-rated vision (with or without glasses). Responses of regular or poor were classified as impaired. | 1 = Regular/poor vision  0 = Good/very good/excellent vision |
|  | **C.44 Hearing.**  Self-rated hearing (with or without a hearing aid).  Responses of regular or poor were classified as impaired. | 1 = Regular/poor hearing  0 = Good/very good/excellent hearing |
|  | **Composite sensory impairment.**  Vision and hearing indicators were combined. | 1 = Impairment in either hearing or vision  0 = No impairment in both domains |
| **Vitality** | **C.64 Weight loss.**  Self-reported weight loss ≥5 kg during the previous two years. | 1 = Weight loss present  0 = No weight loss |
|  | **C.70 Reduced food intake.**  Participants reported eating less because of loss of appetite, digestive problems, or chewing/swallowing difficulties during the previous two years. | 1 = Reduced food intake  0 = No |
|  | **Composite vitality impairment.**  Weight loss and reduced food intake indicators were combined. | 1 = Weight loss and/or reduced food intake  0 = Neither condition present |
| **Mobility** | **H.1 Walking several blocks.**  Self-reported difficulty walking several blocks because of a health problem. | 1 = Difficulty present  0 = No difficulty |
|  | **H.5 Rising from a chair.**  Self-reported difficulty getting up from a chair after sitting for long periods because of a health problem. | 1 = Difficulty present  0 = No difficulty |
|  | **H.6 Climbing stairs.**  Self-reported difficulty climbing several flights of stairs without resting because of a health problem. | 1 = Difficulty present  0 = No difficulty |
|  | **Composite locomotor impairment.**  Walking, chair-rise, and stair-climbing difficulties were combined. | 1 = Difficulty in any activity  0 = No difficulty in all activities |
