# Supplementary material for: Social vulnerability and health determinants of Intrinsic Capacity in Mexican older adults: Evidence from the Mexican Health and Aging Study round 2021
Source: PLoS One. 2026 Aug 7;21(8):e0353903. doi: 10.1371/journal.pone.0353903 (PMC13450843; doi:10.1371/journal.pone.0353903)
Supplement: Supplementary file 3 [file pone.0353903.s004.docx]

**S2 Table.** Sensitivity analysis excluding employment status from the Social Vulnerability Index

| **Variable** | **β coefficient** | **95% CI** | **p-value** |
| --- | --- | --- | --- |
| Social Vulnerability Index (SVI) |  |  |  |
| Low vulnerability (reference) | 0.00 | — | — |
| Intermediate vulnerability | −0.202 | −0.237, −0.168 | <0.001 |
| High vulnerability | −0.377 | −0.420, −0.334 | <0.001 |
| SVI score | −0.154 | −0.168, −0.140 | <0.001 |
| Age group (years) |  |  |  |
| 50–59 (reference) | 0.00 | — | — |
| 60–69 | −0.134 | −0.166, −0.101 | <0.001 |
| 70–79 | −0.459 | −0.496, −0.422 | <0.001 |
| ≥80 | −0.909 | −0.964, −0.854 | <0.001 |
| Female sex | −0.127 | −0.158, −0.096 | <0.001 |
| Poor self-rated health | −0.104 | −0.136, −0.071 | <0.001 |
| History of falls | −0.259 | −0.289, −0.230 | <0.001 |
| Multimorbidity | −0.405 | −0.439, −0.371 | <0.001 |
| Sleep disturbances | −0.533 | −0.563, −0.503 | <0.001 |
| Current alcohol consumption | 0.103 | 0.071, 0.134 | <0.001 |
| Smoking (current or former) | −0.060 | −0.090, −0.029 | <0.001 |

**Abbreviations:** CI, confidence interval.
