# Supplementary material for: Social vulnerability and health determinants of Intrinsic Capacity in Mexican older adults: Evidence from the Mexican Health and Aging Study round 2021
Source: PLoS One. 2026 Aug 7;21(8):e0353903. doi: 10.1371/journal.pone.0353903 (PMC13450843; doi:10.1371/journal.pone.0353903)
Supplement: Supplementary file 4 [file pone.0353903.s005.docx]

**S3 Table**. Variance Inflation Factors (VIFs) for covariates included in multivariable regression models

| **Variable** | **VIF** |
| --- | --- |
| Age group (60–69 years) | 1.47 |
| Age group (70–79 years) | 1.78 |
| Age group (≥80 years) | 1.61 |
| Female sex | 1.55 |
| Widowed | 1.28 |
| Single/divorced/separated | 1.08 |
| Low educational attainment | 1.24 |
| Not currently employed | 1.44 |
| No pension receipt | 1.17 |
| Food insecurity | 1.05 |
| No health-care affiliation | 1.09 |
| Poor self-rated health | 1.04 |
| History of falls | 1.06 |
| Multimorbidity | 1.11 |
| Sleep disturbances | 1.08 |
| Current alcohol consumption | 1.15 |
| Smoking (current or former) | 1.21 |

- Mean VIF = 1.26
- All VIF values were below commonly accepted thresholds, indicating no evidence of problematic multicollinearity.
