# Supplementary material for: Social vulnerability and health determinants of Intrinsic Capacity in Mexican older adults: Evidence from the Mexican Health and Aging Study round 2021
Source: PLoS One. 2026 Aug 7;21(8):e0353903. doi: 10.1371/journal.pone.0353903 (PMC13450843; doi:10.1371/journal.pone.0353903)
Supplement: Supplementary file 5 [file pone.0353903.s006.docx]

**Social Vulnerability and Health Determinants of Intrinsic Capacity in Mexican Older Adults: Evidence from the Mexican Health and Aging Study round 2021.**

The authors address an interesting topic by examining intrinsic capacity through the construction of an index and its association with social and health variables. However, the manuscript presents several conceptual inconsistencies, as well as methodological and discussion limitations. Therefore, I recommend a major revision, and I provide the following comments to strengthen the academic quality of the work.

Introduction

The authors should include a clear conceptual definition of social vulnerability, as well as explain how this concept is linked to the social determinants of health (SDH) framework.

Additionally, the information presented in lines 122-124 is unclear and should be rewritten to improve clarity.

The research objective is not clearly stated. Considering the design of the MHAS and the use of only the 2021 wave, the term “evaluate” is not appropriate. Furthermore, the expressions “key SDH” and “graded association” require clearer definitions.

The introduction is framed using a longitudinal perspective; however, the analysis relies on a single wave of the MHAS. This creates an inconsistency between the conceptual and statistical approaches.

Moreover, the main focus of the study is the analysis of SDH associated with IC, which implies statistical associations rather than measures of inequality. Nonetheless, several parts of the introduction refer to inequalities in IC related to the differential impact of SDH. This inequality-based perspective does not contribute clearly to the stated objective and should either be properly justified or removed.

Methodology

Although the MHAS 2024 wave has already been released, I understand that the manuscript may have been submitted prior to its availability. Therefore, the information in lines 152-155 is unnecessary and must be removed.

Given the importance of constructing the IC index using MHAS data, the authors should provide a supplementary file detailing all questions, variables, and procedures used to generate the index. This will improve transparency and reproducibility.

There is a conceptual issue that may also lead to statistical problems in the construction of the social vulnerability index. This index includes variables that are also used as covariates (e.g., education, employment status, healthcare affiliation), which may introduce multicollinearity in the regression models. The inclusion of this index should be reconsidered, especially since the concept of social vulnerability is not clearly defined in the introduction nor properly integrated into the SDH framework throughout the manuscript.

The regression models lack coherence. The use of OR and coefficients, along with different categorizations of variables, creates confusion regarding the main analytical approach. Moreover, the results from these models appear highly similar. Therefore, a stronger justification is needed for using multiple modeling strategies beyond simple comparison. As a recommendation, selecting a single, well-justified model would improve clarity and focus.

Results

The results should be revised in light of the methodological concerns mentioned above.

There is an inconsistency between the sample size reported in the text and in Figure 1. These values should be carefully verified.

Since the authors applied sample weights, it is necessary to report weighted (expanded) estimates throughout the results.

As previously noted, the inclusion of multiple regression models (ORs vs. coefficients, different categorizations) unnecessarily lengthens the manuscript without adding substantial value, given the similarity of the findings. The analysis should either be simplified or more strongly justified.

Additionally, post-estimation diagnostics, such as VIF, should be reported.

There is also confusion regarding Supplementary Figure 1, which is neither clearly presented nor referenced in the manuscript.

Discussion

The main purpose of this section is to contrast the findings with national and international evidence, highlighting similarities, differences, and possible explanations. However, in its current form, the discussion largely repeats the results using different wording and provides general statements without deeper analytical development. The authors are encouraged to develop a more critical and in-depth discussion.

Furthermore, the discussion should explicitly address the implications of the findings for clinical practice and public policy, as well as the main contributions of the study.

There is also a lack of consistency between the conceptual framework presented in the introduction and the discussion of results. Concepts such as SDH and social vulnerability are not adequately revisited or interpreted in light of the findings.

The authors should acknowledge as a limitation that, although the MHAS allows for longitudinal analysis, this study is restricted to the 2021 wave. A justification for this choice should also be provided.

Another limitation that should be explicitly stated is that the IC index constructed in this study is not directly comparable with those used in other research.

Finally, the content in lines 496-500 is repetitive and should be revised or removed.
