# Supplementary material for: Social vulnerability and health determinants of Intrinsic Capacity in Mexican older adults: Evidence from the Mexican Health and Aging Study round 2021
Source: PLoS One. 2026 Aug 7;21(8):e0353903. doi: 10.1371/journal.pone.0353903 (PMC13450843; doi:10.1371/journal.pone.0353903)
Supplement: Peer review history

# Peer review: Decision Letter 0

**DOI:** 10.1371/journal.pone.0353903.r001

**Authors:** Guillermo Salinas-Escudero

30 Apr 2026

PONE-D-26-15851

Social Vulnerability and Health Determinants of Intrinsic Capacity in Mexican Older Adults: Evidence from the Mexican Health and Aging Study round 2021.

PLOS One

Dear Dr. Aguilar Navarro,

Thank you for submitting your manuscript to PLOS ONE. After careful consideration, we feel that it has merit but does not fully meet PLOS ONE’s publication criteria as it currently stands. Therefore, we invite you to submit a revised version of the manuscript that addresses the points raised during the review process.

==============================

The work requires major changes to meet the methodological and quality requirements of the journal Plos One, therefore we request that you work on a new version of the manuscript considering the recommendations made by the reviewers.

==============================

Please submit your revised manuscript by Jun 14 2026 11:59PM. If you will need more time than this to complete your revisions, please reply to this message or contact the journal office at plosone@plos.org. When you're ready to submit your revision, log on to https://www.editorialmanager.com/pone/ and select the 'Submissions Needing Revision' folder to locate your manuscript file.

Please include the following items when submitting your revised manuscript:

- A letter that responds to each point raised by the academic editor and reviewer(s). You should upload this letter as a separate file labeled 'Response to Reviewers'.
- A marked-up copy of your manuscript that highlights changes made to the original version. You should upload this as a separate file labeled 'Revised Manuscript with Track Changes'.
- An unmarked version of your revised paper without tracked changes. You should upload this as a separate file labeled 'Manuscript'.

If you would like to make changes to your financial disclosure, please include your updated statement in your cover letter. Guidelines for resubmitting your figure files are available below the reviewer comments at the end of this letter.

If applicable, we recommend that you deposit your laboratory protocols in protocols.io to enhance the reproducibility of your results. Protocols.io assigns your protocol its own identifier (DOI) so that it can be cited independently in the future. For instructions see: https://journals.plos.org/plosone/s/submission-guidelines#loc-laboratory-protocols. Additionally, PLOS ONE offers an option for publishing peer-reviewed Lab Protocol articles, which describe protocols hosted on protocols.io. Read more information on sharing protocols at https://plos.org/protocols?utm_medium=editorial-email&utm_source=authorletters&utm_campaign=protocols.

As the corresponding author, your ORCID iD is verified in the submission system and will appear in the published article. PLOS supports the use of ORCID, and we encourage all coauthors to register for an ORCID iD and use it as well. Please encourage your coauthors to verify their ORCID iD within the submission system before final acceptance, as unverified ORCID iDs will not appear in the published article. *Only*  the individual author can complete the verification step; PLOS staff *cannot*  verify ORCID iDs on behalf of authors.

We look forward to receiving your revised manuscript.

Kind regards,

Guillermo Salinas-Escudero, PhD. MsC.

Academic Editor

PLOS One

Journal Requirements:

When submitting your revision, we need you to address these additional requirements.

1. Please ensure that your manuscript meets PLOS ONE's style requirements, including those for file naming. The PLOS ONE style templates can be found at

https://journals.plos.org/plosone/s/file?id=wjVg/PLOSOne_formatting_sample_main_body.pdf and

https://journals.plos.org/plosone/s/file?id=ba62/PLOSOne_formatting_sample_title_authors_affiliations.pdf

2. Thank you for uploading your study's underlying data set. Unfortunately, the repository you have noted in your Data Availability statement does not qualify as an acceptable data repository according to PLOS's standards.

At this time, please upload the minimal data set necessary to replicate your study's findings to a stable, public repository (such as figshare or Dryad) and provide us with the relevant URLs, DOIs, or accession numbers that may be used to access these data. For a list of recommended repositories and additional information on PLOS standards for data deposition, please see https://journals.plos.org/plosone/s/recommended-repositories.

3. We notice that your supplementary figures are uploaded with the file type 'Figure'. Please amend the file type to 'Supporting Information'. Please ensure that each Supporting Information file has a legend listed in the manuscript after the references list.

4. We notice that your supplementary tables are included in the manuscript file. Please remove them and upload them with the file type 'Supporting Information'. Please ensure that each Supporting Information file has a legend listed in the manuscript after the references list.

5. If the reviewer comments include a recommendation to cite specific previously published works, please review and evaluate these publications to determine whether they are relevant and should be cited. There is no requirement to cite these works unless the editor has indicated otherwise.

[Note: HTML markup is below. Please do not edit.]

Reviewers' comments:

Reviewer's Responses to Questions

**Comments to the Author**

1. Is the manuscript technically sound, and do the data support the conclusions?

The manuscript must describe a technically sound piece of scientific research with data that supports the conclusions. Experiments must have been conducted rigorously, with appropriate controls, replication, and sample sizes. The conclusions must be drawn appropriately based on the data presented.

Reviewer #1: Partly

Reviewer #2: No

**********

2. Has the statistical analysis been performed appropriately and rigorously?

Reviewer #1: Yes

Reviewer #2: No

**********

3. Have the authors made all data underlying the findings in their manuscript fully available?

The PLOS Data policy requires authors to make all data underlying the findings described in their manuscript fully available without restriction, with rare exception (please refer to the Data Availability Statement in the manuscript PDF file). The data should be provided as part of the manuscript or its supporting information, or deposited to a public repository. For example, in addition to summary statistics, the data points behind means, medians and variance measures should be available. If there are restrictions on publicly sharing data—e.g. participant privacy or use of data from a third party—those must be specified.

Reviewer #1: Yes

Reviewer #2: No

**********

4. Is the manuscript presented in an intelligible fashion and written in standard English?

PLOS ONE does not copyedit accepted manuscripts, so the language in submitted articles must be clear, correct, and unambiguous. Any typographical or grammatical errors should be corrected at revision, so please note any specific errors here.

Reviewer #1: Yes

Reviewer #2: Yes

**********

5. Review Comments to the Author

Please use the space provided to explain your answers to the questions above. You may also include additional comments for the author, including concerns about dual publication, research ethics, or publication ethics. (Please upload your review as an attachment if it exceeds 20,000 characters)

Reviewer #1: The methodology is adequate for a cross-sectional, hypothesis-generating study but has significant flaws that limit causal inference and internal validity for certain domains (notably locomotion and SVI). The authors have done a commendable job operationalizing IC using CFA and constructing a cumulative SVI. However, the extremely high prevalence of locomotor impairment (96.8%) threatens domain discrimination, and the contradictory findings for the SVI (continuous vs. categorical) and alcohol consumption raise concerns about model specification and residual confounding.

Construction issues:

SVI includes “living alone.” Living alone is not universally a deficit in older adults, as it may indicate independence and resources. Social isolation would be a better choice.

SVI includes “not currently employed” but for adults ≥70 years, this is normative, not a social vulnerability deficit.

Cross-Sectional Design vs. Causal Language

There is a problem as authors state objectives like “identify early vulnerability” and interpret associations directionally (e.g., “IC predicts,” “social disadvantage shapes IC”). However, with cross-sectional data from a single wave (2021), temporality cannot be established. The Discussion repeatedly uses causal language (e.g., “smoking’s role in accelerating functional aging”), which is not supported by the design.

Operationalization of IC Domains – Potential Misalignment with WHO ICOPE

While the authors state they follow the WHO ICOPE framework, several domain proxies are questionable, in particular the locomotor domain: Difficulty with 4 activities (walking blocks, stairs, home maintenance, rising from chair) is overly sensitive: 96.8% prevalence of impairment. This is likely ceiling effect, rendering the domain almost useless for discriminating among community-dwelling adults. “Home maintenance/repairs” is heavily influenced by gender roles and housing conditions, not pure locomotion.

Reviewer #2: The authors address an interesting topic by examining intrinsic capacity through the construction of an index and its association with social and health variables. However, the manuscript presents several conceptual inconsistencies, as well as methodological and discussion limitations. Therefore, I recommend a major revision, and I provide the following comments to strengthen the academic quality of the work.

**********

6. PLOS authors have the option to publish the peer review history of their article (what does this mean?). If published, this will include your full peer review and any attached files.

If you choose “no”, your identity will remain anonymous but your review may still be made public.

**Do you want your identity to be public for this peer review?**  For information about this choice, including consent withdrawal, please see our Privacy Policy.

Reviewer #1: **Yes:** Luis Miguel Gutierrez Robledo

Reviewer #2: No

**********

[NOTE: If reviewer comments were submitted as an attachment file, they will be attached to this email and accessible via the submission site. Please log into your account, locate the manuscript record, and check for the action link "View Attachments". If this link does not appear, there are no attachment files.]

To ensure your figures meet our technical requirements, please review our figure guidelines: https://journals.plos.org/plosone/s/figures

You may also use PLOS’s free figure tool, NAAS, to help you prepare publication quality figures: https://journals.plos.org/plosone/s/figures#loc-tools-for-figure-preparation.

NAAS will assess whether your figures meet our technical requirements by comparing each figure against our figure specifications.

Attachment Submitted filename: Review.docx

# Author comment: Author response to Decision Letter 1

**DOI:** 10.1371/journal.pone.0353903.r002

12 Jun 2026

Mexico City June 11 2026

Manuscript Title: Social Vulnerability and Health Determinants of Intrinsic Capacity in Mexican Older Adults: Evidence from the Mexican Health and Aging Study round 2021.

Manuscript ID: PONE-D-26-15851

Dear Reviewers,

We sincerely thank you for your thorough evaluation of our manuscript and for the constructive comments and suggestions. We have carefully revised the manuscript accordingly. Below, we provide a detailed, point-by-point response to each comment. All changes have been incorporated into the revised manuscript and are highlighted using track changes.

Comments from Reviewer #1

1. Comment 1:The methodology is adequate for a cross-sectional, hypothesis-generating study but has significant flaws that limit causal inference and internal validity for certain domains (notably locomotion and SVI). The authors have done a commendable job operationalizing IC using CFA and constructing a cumulative SVI. However, the extremely high prevalence of locomotor impairment (96.8%) threatens domain discrimination, and the contradictory findings for the SVI (continuous vs. categorical) and alcohol consumption raise concerns about model specification and residual confounding.

a. Response: We thank the reviewer for these valuable comments.

Regarding the locomotion domain, we re-examined its operationalization and found that the household maintenance item was highly prevalent and contributed substantially to the very high prevalence of locomotor impairment. Additionally, this indicator showed the weakest contribution in the CFA. Therefore, we revised the locomotion domain by excluding this item and retaining measures more directly related to physical mobility (walking several blocks, climbing stairs, and rising from a chair).

Regarding the SVI, we acknowledge the different findings obtained when modeling the index as a continuous versus categorical variable. We believe this reflects a potential non-linear association between social vulnerability and intrinsic capacity. We have clarified this interpretation and acknowledged it as a study limitation.

Regarding alcohol consumption, the MHAS only captures current alcohol use and does not provide information on quantity, frequency, lifetime exposure, or former drinking status. Therefore, residual confounding and a potential “healthy drinker” effect cannot be excluded. We have expanded the limitations section accordingly.

Finally, we further emphasize that the cross-sectional design precludes causal inference and that the findings should be interpreted as associations useful for hypothesis generation.

2. Comment 2:

a. Construction issues:

i. SVI includes “living alone.” Living alone is not universally a deficit in older adults, as it may indicate independence and resources. Social isolation would be a better choice.

ii. SVI includes “not currently employed” but for adults ≥70 years, this is normative, not a social vulnerability deficit.

b. Response: We thank the reviewer for this comment. We agree that living alone does not universally represent a deficit and may, in some cases, reflect autonomy and independence. However, evidence from both Mexican and international studies has linked living alone to adverse outcomes in older adults. In Mexico, Franco-Alvarez et al. (2007) identified living alone as a determinant of malnutrition risk among community-dwelling older adults, and Rodríguez-Tadeo et al. (2012) reported that living alone remained independently associated with nutritional risk after multivariable adjustment.Internationally, Abell and Steptoe (2021) found that living alone was associated with a 20% higher mortality risk among older adults. Furthermore, living alone was explicitly included as a component of the Social Vulnerability Index proposed by Andrew, Mitnitski, and Rockwood (2008). Therefore, although living alone should not be interpreted as a vulnerability deficit per se, we considered it a meaningful indicator of potential social vulnerability and retained it as one component of the multidimensional SVI.

Regarding employment status, we agree that retirement and labor-force exit are common transitions in later life and may not uniformly reflect vulnerability across all older adults. To address this concern, we conducted a sensitivity analysis excluding employment status from the SVI and repeated all SVI analyses. The association between social vulnerability and intrinsic capacity remained significant. Compared with the lowest vulnerability tertile, intermediate vulnerability was associated with lower intrinsic capacity (β = −0.202; 95% CI: −0.237 to −0.168; p < 0.001), and high vulnerability showed an even stronger association (β = −0.377; 95% CI: −0.420 to −0.334; p < 0.001). Similarly, the continuous SVI excluding employment status remained significantly associated with lower intrinsic capacity (β = −0.154; 95% CI: −0.168 to −0.140; p < 0.001). These results are presented in Supplementary Table S2 and indicate that the association between social vulnerability and intrinsic capacity was not driven by the inclusion of employment status in the index.

3. Comment 3:

The manuscript uses potentially causal language (e.g., “identify early vulnerability,” “IC predicts,” “social disadvantage shapes IC”) despite the cross-sectional design, which does not allow for establishing temporality.

a. Response: We thank the reviewer for this important observation. We agree that the cross-sectional design does not allow the establishment of temporality or causal inference. Accordingly, we carefully reviewed the manuscript and revised statements that could be interpreted as causal. Terms such as “predict,” “shape,” “contribute to,” and “identify early vulnerability” were replaced with more appropriate language emphasizing associations, correlates, or relationships. We have also reinforced in the Discussion and Conclusion sections that the findings should be interpreted as cross-sectional associations rather than causal effects.These changes have been incorporated in lines 69, 70, 89, 90, 120, 521 of the revised manuscript.

4. Comment 4:

The Discussion includes causal interpretations (e.g., “smoking’s role in accelerating functional aging”) that are not supported by the study design.

a. Response: We thank the reviewer for this observation. We agree that the cross-sectional design does not support causal interpretations regarding the effects of smoking or other factors on intrinsic capacity. Accordingly, we revised the Discussion to avoid causal language and to emphasize associations. Specifically, statements referring to smoking as “accelerating functional aging” were rephrased to indicate that smoking was associated with lower intrinsic capacity and more extensive impairment in our study population. Similar revisions were made where necessary to ensure that all findings are interpreted as associations rather than causal effects. These changes have been incorporated in lines 478-479 of the revised manuscript.

5. Comment 9:

Operationalization of IC Domains – Potential Misalignment with WHO ICOPE

a. While the authors state they follow the WHO ICOPE framework, several domain proxies are questionable, in particular the locomotor domain: Difficulty with 4 activities (walking blocks, stairs, home maintenance, rising from chair) is overly sensitive: 96.8% prevalence of impairment. This is likely ceiling effect, rendering the domain almost useless for discriminating among community-dwelling adults. “Home maintenance/repairs” is heavily influenced by gender roles and housing conditions, not pure locomotion.

b. Response: We appreciate the reviewer’s observation. We agreed that the original operationalization of the locomotion domain showed limited discriminatory capacity, as the prevalence of impairment reached 96.8%. After further evaluation, we identified the household maintenance/repair item as the primary contributor to this issue, as it may reflect household roles and environmental factors in addition to mobility limitations. Accordingly, we excluded this item and redefined the locomotion domain using indicators more directly related to mobility (walking several blocks, climbing stairs, and rising from a chair). This modification reduced the prevalence of locomotor impairment from 96.8% to 43.1%, substantially improving domain discrimination. All analyses were repeated using the revised definition, and the main findings remained materially unchanged, supporting the robustness of the results. The revised operationalization has been updated throughout the manuscript.

Comments from Reviewer #2

Introduction

1. Comment 1: The authors should include a clear conceptual definition of social vulnerability, as well as explain how this concept is linked to the social determinants of health (SDH) framework.

a. Response: We thank the reviewer for this valuable observation. In response, we added a clear conceptual definition of social vulnerability and further clarified its relationship with the Social Determinants of Health (SDH) framework. These additions have been incorporated in lines 115–124 of the revised manuscript.

2. Comment 2: Additionally, the information presented in lines 122-124 is unclear and should be rewritten to improve clarity.

a. Response: We thank the reviewer for this comment. The text has been revised to improve clarity and readability, and we hope the revised version provides a clearer presentation of the intended concept. These changes have been incorporated in lines 130–136 of the revised manuscript.

3. Comment 3: The research objective is not clearly stated. Considering the design of the MHAS and the use of only the 2021 wave, the term “evaluate” is not appropriate. Furthermore, the expressions “key SDH” and “graded association” require clearer definitions.

a. Response: We appreciate this observation. To improve clarity, we revised the study objective to better align with the cross-sectional design, clarified the social determinants of health considered in the analyses, and more clearly described the progressive association observed between higher levels of social vulnerability and lower intrinsic capacity. These changes have been incorporated in lines 141, 317 y 372 of the revised manuscript.

4. Comment 4: The introduction is framed using a longitudinal perspective; however, the analysis relies on a single wave of the MHAS. This creates an inconsistency between the conceptual and statistical approaches.

a. Response: We thank the reviewer for this important observation. We agree that the previous version of the introduction contained language that could imply a longitudinal perspective and was not fully aligned with the cross-sectional nature of our analysis based on a single wave of the MHAS. In response, we revised the introduction, including the wording and supporting evidence, to adopt a more cross-sectional framework and avoid language suggestive of temporal trajectories or causal pathways.

5. Comment 5: Moreover, the main focus of the study is the analysis of SDH associated with IC, which implies statistical associations rather than measures of inequality. Nonetheless, several parts of the introduction refer to inequalities in IC related to the differential impact of SDH. This inequality-based perspective does not contribute clearly to the stated objective and should either be properly justified or removed.

a. Response: the change was done

Methodology

1. Comment 9:

The information in lines 152–155 regarding the MHAS 2024 wave is unnecessary and should be removed.

a. Response: We appreciate the reviewer’s observation. The statement referring to the 2024 MHAS wave was removed, as it was not essential to the description of the study design or data source. The Methods section has been revised accordingly.

2. Comment 10:

Given the importance of constructing the IC index using MHAS data, a supplementary file detailing all questions, variables, and procedures used to generate the index should be provided to improve transparency and reproducibility.

a. Response: Thank you for this comment. We agree that detailed documentation of the IC index construction is essential for transparency and reproducibility. Accordingly, we have added a supplementary table describing the MHAS 2021 variables, operationalization procedures, and coding rules used to derive each IC domain and the overall IC index.

3. Comment 11:

There is a conceptual issue that may also lead to statistical problems in the construction of the social vulnerability index, as it includes variables that are also used as covariates (e.g., education, employment status, healthcare affiliation), which may introduce multicollinearity in the regression models.

a. Response: We appreciate the reviewer’s observation. We would like to clarify that the individual social variables included in the Social Vulnerability Index (SVI) were not entered simultaneously with the SVI in the same regression models. Tables 2–4 examined the associations of individual social and health-related factors with intrinsic capacity, whereas Table 5 evaluated the association between the composite SVI and intrinsic capacity. Therefore, the SVI components and the SVI itself were not modeled concurrently, minimizing concerns regarding multicollinearity.

To further address this concern, we assessed multicollinearity among covariates included in the regression models using variance inflation factors (VIFs). All VIF values were low (range: 1.04–1.78; mean VIF = 1.26), indicating that multicollinearity was not a concern (Supplementary Table S3).

In addition, we conducted a sensitivity analysis excluding employment status from the SVI and repeated all SVI analyses. The results remained materially unchanged (Supplementary Table S2), supporting the robustness of the findings and indicating that the observed associations were not driven by overlap among individual SVI components.

4. Comment 12:

The inclusion of this index should be reconsidered, especially since the concept of social vulnerability is not clearly defined in the introduction nor properly integrated into the SDH framework throughout the manuscript.

a. Response: We appreciate the reviewer’s observation. In response, we strengthened the conceptual framework presented in the Introduction by incorporating explicit definitions of both Social Determinants of Health (SDH) and social vulnerability, and by clarifying the relationship between these concepts. We also expanded the rationale for including the Social Vulnerability Index (SVI) by incorporating recent literature on social vulnerability indices and the social determinants of intrinsic capacity (Mah et al., 2023; Zhao et al., 2024). These revisions were incorporated into the Introduction (approximately lines 115–130 of the revised manuscript).

To further improve clarity, we developed a conceptual framework illustrating the relationship between SDH, social vulnerability, and intrinsic capacity (Supplementary Figure S1). This figure highlights how individual social determinants contribute to cumulative social vulnerability, which in turn is associated with intrinsic capacity outcomes. Together, these revisions provide a clearer theoretical justification for the inclusion of the SVI in the present study.

5. Comment 13:

The regression models lack coherence. The use of odds ratios and coefficients, along with different categorizations of variables, creates confusion regarding the main analytical approach.

a. Response: We appreciate the reviewer’s observation. To improve clarity and coherence, we revised the Statistical Analysis section (lines 259–282) to more clearly describe the rationale for each analytical approach. Specifically, we clarified that ordinal logistic regression was used for the ordered IC impairment groups, linear regression for the continuous latent IC score, multinomial logistic regression for IC tertiles, and linear regression models for the association between the Social Vulnerability Index (SVI) and the continuous IC score. We also explicitly stated the corresponding effect measures reported for each model (ORs, RRRs, and β coefficients). These revisions were intended to clarify that the different models reflect complementary operationalizations of intrinsic

Attachment Submitted filename: Response to Reviewers.pdf

# Peer review: Decision Letter 1

**DOI:** 10.1371/journal.pone.0353903.r003

**Authors:** Guillermo Salinas-Escudero

1 Jul 2026

<p>Social Vulnerability and Health Determinants of Intrinsic Capacity in Mexican Older Adults: Evidence from the Mexican Health and Aging Study round 2021.

PONE-D-26-15851R1

Dear Dr. Aguilar Navarro,

We’re pleased to inform you that your manuscript has been judged scientifically suitable for publication and will be formally accepted for publication once it meets all outstanding technical requirements.

Within one week, you’ll receive an e-mail detailing the required amendments. When these have been addressed, you’ll receive a formal acceptance letter and your manuscript will be scheduled for publication.

An invoice will be generated when your article is formally accepted. Please note, if your institution has a publishing partnership with PLOS and your article meets the relevant criteria, all or part of your publication costs will be covered. Please make sure your user information is up-to-date by logging into Editorial Manager at Editorial Manager® and clicking the ‘Update My Information' link at the top of the page. For questions related to billing, please contact billing support.

If your institution or institutions have a press office, please notify them about your upcoming paper to help maximize its impact. If they’ll be preparing press materials, please inform our press team as soon as possible -- no later than 48 hours after receiving the formal acceptance. Your manuscript will remain under strict press embargo until 2 pm Eastern Time on the date of publication. For more information, please contact onepress@plos.org.

Kind regards,

Guillermo Salinas-Escudero, PhD. MsC.

Academic Editor

PLOS One

Additional Editor Comments (optional):

Reviewers' comments:

Reviewer's Responses to Questions

**Comments to the Author**

1. If the authors have adequately addressed your comments raised in a previous round of review and you feel that this manuscript is now acceptable for publication, you may indicate that here to bypass the “Comments to the Author” section, enter your conflict of interest statement in the “Confidential to Editor” section, and submit your "Accept" recommendation.

Reviewer #1: All comments have been addressed

Reviewer #2: All comments have been addressed

**********

2. Is the manuscript technically sound, and do the data support the conclusions?

The manuscript must describe a technically sound piece of scientific research with data that supports the conclusions. Experiments must have been conducted rigorously, with appropriate controls, replication, and sample sizes. The conclusions must be drawn appropriately based on the data presented.

Reviewer #1: Yes

Reviewer #2: Yes

**********

3. Has the statistical analysis been performed appropriately and rigorously?

Reviewer #1: Yes

Reviewer #2: Yes

**********

4. Have the authors made all data underlying the findings in their manuscript fully available?

The PLOS Data policy requires authors to make all data underlying the findings described in their manuscript fully available without restriction, with rare exception (please refer to the Data Availability Statement in the manuscript PDF file). The data should be provided as part of the manuscript or its supporting information, or deposited to a public repository. For example, in addition to summary statistics, the data points behind means, medians and variance measures should be available. If there are restrictions on publicly sharing data—e.g. participant privacy or use of data from a third party—those must be specified.

Reviewer #1: Yes

Reviewer #2: Yes

**********

5. Is the manuscript presented in an intelligible fashion and written in standard English?

PLOS ONE does not copyedit accepted manuscripts, so the language in submitted articles must be clear, correct, and unambiguous. Any typographical or grammatical errors should be corrected at revision, so please note any specific errors here.

Reviewer #1: Yes

Reviewer #2: Yes

**********

6. Review Comments to the Author

Please use the space provided to explain your answers to the questions above. You may also include additional comments for the author, including concerns about dual publication, research ethics, or publication ethics. (Please upload your review as an attachment if it exceeds 20,000 characters)

Reviewer #1: (No Response)

Reviewer #2: The manuscript has improved substantially, considering the comments provided in a previous review. I don't have additional observations.

**********

7. PLOS authors have the option to publish the peer review history of their article (what does this mean?). If published, this will include your full peer review and any attached files.

If you choose “no”, your identity will remain anonymous but your review may still be made public.

**Do you want your identity to be public for this peer review?**  For information about this choice, including consent withdrawal, please see our Privacy Policy.

Reviewer #1: No

Reviewer #2: No

**********

# Editor report: Acceptance letter

**DOI:** 10.1371/journal.pone.0353903.r004

**Authors:** Guillermo Salinas-Escudero

PONE-D-26-15851R1

PLOS One

Dear Dr. Aguilar Navarro,

I'm pleased to inform you that your manuscript has been deemed suitable for publication in PLOS One. Congratulations! Your manuscript is now being handed over to our production team.

At this stage, our production department will prepare your paper for publication. This includes ensuring the following:

* All references, tables, and figures are properly cited

* All relevant supporting information is included in the manuscript submission,

* There are no issues that prevent the paper from being properly typeset

You will receive further instructions from the production team, including instructions on how to review your proof when it is ready. Please keep in mind that we are working through a large volume of accepted articles, so please give us a few days to review your paper and let you know the next and final steps.

Lastly, if your institution or institutions have a press office, please let them know about your upcoming paper now to help maximize its impact. If they'll be preparing press materials, please inform our press team within the next 48 hours. Your manuscript will remain under strict press embargo until 2 pm Eastern Time on the date of publication. For more information, please contact onepress@plos.org.

You will receive an invoice from PLOS for your publication fee after your manuscript has reached the completed accept phase. If you receive an email requesting payment before acceptance or for any other service, this may be a phishing scheme. Learn how to identify phishing emails and protect your accounts at https://explore.plos.org/phishing.

If we can help with anything else, please email us at customercare@plos.org.

Thank you for submitting your work to PLOS ONE and supporting open access.

Kind regards,

PLOS ONE Editorial Office Staff

on behalf of

Dr. Guillermo Salinas-Escudero

Academic Editor

PLOS One
